# Secreted Frizzled‐Related Protein 2 (SFRP2) Induces Follistatin‐Like 1 (FSTL1) to Regulate Dihydrotestosterone (DHT)‐Induced Dermal Papilla Cell Mitochondrial Dysfunction and Senescence

**DOI:** 10.1111/acel.70666

**Published:** 2026-09-04

**Authors:** Youming Huang, Qiong Bian, Yeyu Shen, Xiaoxia Ding, Yan Teng, Danfeng Xu, Xianhong Yang, Yibin Fan

**Affiliations:** ^1^ Department of Dermatology Center for Plastic and Reconstructive Surgery, Zhejiang Provincial People's Hospital, Affiliated People's Hospital, Hangzhou Medical College Hangzhou China

**Keywords:** cell senescence, follistatin‐like 1 (FSTL1), human dermal papilla cells (DPCs), mitochondrial dysfunction, oxidative stress, secreted frizzled‐related protein 2 (SFRP2)

## Abstract

Androgenetic alopecia (AGA) is the most common form of non‐scarring hair loss, driven by genetic factors and increased sensitivity of scalp hair follicles to dihydrotestosterone (DHT), which causes progressive miniaturization of dermal papilla cells and shortens the hair growth phase. The precise molecular pathogenesis of AGA remains incompletely understood. The study aimed to elucidate the regulatory mechanism between secreted frizzled‐related protein 2 (SFRP2) and follistatin‐like 1 (FSTL1), explore their impact on DHT‐induced mitochondrial dysfunction and senescence in dermal papilla cells (DPCs), elucidate the effect of the SFRP2–FSTL1 axis on oxidative stress‐related DPCs changes, and identify new therapeutic targets for AGA treatment. In the study, SFRP2 was highly expressed in the DPCs of AGA patients. Knocking down SFRP2 improved hair regeneration and follicle morphology within AGA model mice, promoting proliferation, migration, and invasion of DPCs in vitro. SFRP2 knockdown also alleviated inflammation, senescence, mitochondrial dysfunction, and oxidative stress. SFRP2 was found to bind to FSTL1, thereby promoting FSTL1 protein stability. Knocking down FSTL1 counteracted the negative effects of SFRP2 overexpression in vitro. In conclusion, SFRP2 emerges as a critical regulator in AGA by promoting stability and activity of FSTL1, establishing a novel SFRP2–FSTL1 axis that exacerbates DHT‐driven pathogenic changes in DPCs. These findings identify SFRP2 and FSTL1 as key mediators of androgen‐induced cellular dysfunction and suggest that disrupting this axis could offer a promising therapeutic strategy for slowing or reversing the progression of AGA.

## Graphical abstract

Secreted frizzled‐related protein 2 (SFRP2) emerges as a critical regulator in androgenetic alopecia (AGA) by promoting stability and activity of the follistatin‐like protein FSTL1, establishing a novel SFRP2–FSTL1 axis that exacerbates dihydrotestosterone (DHT)‐driven pathogenic changes in dermal papilla cells (DPCs).

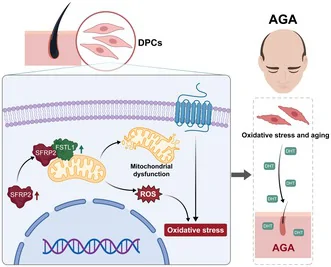

## 1 Introduction

Androgenetic Alopecia (AGA), a chronic and progressive form of hair loss, exhibits a strong association with genetics and fluctuations in androgen levels (Devjani et al. 2023). Dihydrotestosterone (DHT), a specific type of androgen, serves as a crucial factor in the atrophy of hair follicles (Nestor et al. 2021). When present in excessive amounts, DHT can impair the normal growth cycle of hair follicles, resulting in the formation of vellus‐like, tiny hairs as the follicles cease their growth and ultimately result in hair loss (Gupta et al. 2022). Currently, the treatment modalities for AGA primarily encompass drug therapy and hair transplantation (Ly et al. 2023). However, the limited efficacy of drug‐based treatments and the high cost of surgical interventions have posed challenges for individuals affected by this condition. Therefore, conducting in‐depth investigations into AGA pathogenesis and uncovering novel therapeutic drugs hold significant importance for effectively managing this prevalent form of hair loss.

Oxidative stress, cell senescence, and mitochondrial dysfunction are the primary culprits behind hair loss (Deng et al. 2023; Natarelli et al. 2024; Trüeb 2021). Dermal papilla cells (DPCs), a cluster of mesenchymal cells situated in the hair bulb, exert a pivotal effect on the regeneration and cycle regulation of hair follicles (Zhang et al. 2024). They act as a crucial link, providing the essential signals for hair follicle regeneration and dictating the differentiation trajectory of hair follicles (Ceruti et al. 2021; Fusenig et al. 1994). One of the unique functions of DPCs is their ability to induce hair follicles (Taghiabadi et al. 2020; Zheng and Xu 2023). The aging process of DPCs is predominantly triggered by excessive DHT. This, in turn, sets off an inflammatory response within the scalp environment, leading to the release of pro‐inflammatory mediators including interleukin‐6 (IL‐6), IL‐1β and tumor necrosis factor‐α (TNF‐α) (Ma et al. 2022). The overexpression of these molecules could expedite the hair papilla cell aging and demise. Simultaneously, androgen‐induced DPCs aging involves the regulation of multiple genes. There are alterations in the expression levels of aging‐associated genes like senescence‐associated β‐galactosidase (SA‐β‐Gal), p21, and p16, which directly impact the survival of DPCs and hasten the progression of hair loss (Ei et al. 2024; Shin et al. 2021). Mitochondrial dysfunction gives rise to metabolic disorders in DPCs. DPCs within bald hair follicles are subjected to heightened oxidative stress. The elevation of oxidative phosphorylation and reactive oxygen‐free radical levels can result in either apoptosis or premature aging of DPCs (Chew et al. 2022; Natarelli et al. 2024). Ultimately, this leads to the replacement of terminal hair with vellus hair. Although the premature aging of DPCs is closely intertwined with oxidative stress, cell aging, and mitochondrial dysfunction, the underlying mechanism of action remains shrouded in mystery.

Secreted frizzled‐related protein 2 (SFRP2) belongs to the SFRP family, which consists of a cysteine‐rich domain that is similar to the Frizzled protein and can bind to Wnt (van Loon et al. 2021). Transcriptome analysis identified modulators of the Wnt signaling and hypoxia‐inducible factor pathways as potential mediators of androgenetic alopecia (Philpott 2022). Previous studies have described key transcriptome alterations within male AGA hair follicles, and the Wnt pathway inhibitor SFRP2 might exert an important effect on AGA (Liu et al. 2022). SFRP2 can regulate mitochondrial dynamics, accelerate mitochondrial biogenesis, oxidative stress and apoptosis within diabetic cardiomyopathy (Ma et al. 2021). In this research, our bioinformatics analysis showed that FSTL1 is a directly related gene of SFRP2, and FSTL1 is positively associated with mitochondrial function. In vitro, intracellular FSTL1 is localized in mitochondria. FSTL1 activates the mitochondrial electron transport chain, increases the secretion of ATP, a pivotal activator of inflammasomes, and the secretion of IL‐1β.

In summary, we investigated how SFRP2 influences FSTL1 through bioinformatics analysis, and then utilized mouse and cell models to examine further how the SFRP2/FSTL1 axis enhances mitochondrial dysfunction, regulates DPCs aging, and ultimately reverses AGA progression. By analyzing the mechanism of DPCs aging and mitochondrial dysfunction in AGA progression from a new perspective, we provide a novel perspective for the development of therapeutic targets for the treatment of AGA.

## 2 Materials and Methods

### 2.1 Animal Grouping and Treatment

Male C57BL/6 mice (6‐week‐old, *N* = 30) were obtained from Hunan SJA Laboratory Animal Co. Ltd. (Changsha, China). The mice were kept within a Specific Pathogen Free (SPF) animal room (12 h light/dark cycles, 22°C ± 2°C, 55% ± 5% humidity) for 1 week and were given free access to food and water. Mice were randomized into five groups (*N* = 6): Control, DHT, DHT + lv‐shNC, DHT + lv‐shSFRP2#1, DHT + lv‐shSFRP2#2. Before experiments, all the mice were subjected to hair removal by shaving, hair removal cream (Reckitt Benckiser, Jingzhou, China) and rosin & wax depilation. The androgenic alopecia model was prepared via intraperitoneal injection with 200 μL of DHT solution (0.5 mg/mL) at days 1, 3, 5, 7, 17, 19, and 21, whereas the normal controls were given an equal amount of normal saline. The constructed SFRP2 short hairpin RNA (shRNA) lentivirus (lv‐shSFRP2#1 and lv‐shSFRP2#2, 10^9^ TU/mL) was administered to mice via tail vein injection at a volume of 150 μL for two consecutive days. The negative control lentivirus carried a non‐targeting shRNA sequence (lv‐shNC) without known effects on mouse genes and was used to exclude potential non‐specific effects of viral transduction. The dorsal skin of mice was observed and photographed on days 12 and 24 after depilation based on previous studies (Fu et al. 2024; Huang et al. 2026). The study was carried out under the approval of the Laboratory Animal Ethics Committee of Zhejiang Provincial People's Hospital (Approval No.: 2024‐022).

### 2.2 Recording and Scoring of the Mice Back Skin Hair

The skin in the depilated area of each group was observed daily. The dorsal skin of mice was recorded and imaged on days 12 and 24. The skin darkening time and when the hair began to grow (hair regrowth time) were recorded, and the hair growth of mice was scored every 6 days (days 12, 18, 24). Scoring rules: 1 = 0%–20% growth; 2 = 20%–40% growth; 3 = 40%–60% growth; 4 = 60%–80% growth; 5 = 80%–100% growth. On day 24 of the experiment, 3 hairs were collected from the front, middle, and back of the hair removal area, and the average hair length of each group of mice was recorded.

### 2.3 Hematoxylin and Eosin (H&E) Staining and Hair Follicle Count

Skin tissues with hair follicles were obtained from the mouse depilatory area, subjected to an overnight fixation with 4% paraformaldehyde, paraffin‐embedded, and sliced at 5 μm. Slices were then subjected to staining with the H&E kit (G1120, Solarbio, Beijing, China), photographed, and ImageJ was employed to count the hair follicle number.

### 2.4 Cell Culture and Transfection

Human DPCs (CP‐H249, Pricella Biotechnology, Wuhan, China) from the same commercial batch were cultivated within Complete Culture Medium for Human DPCs (CM‐H249, Pricella Biotechnology) in a cell incubator (37°C, 5% CO_2_ and 95% air atmosphere).

SFRP2 overexpression plasmids and plasmids containing short hairpin RNA targeted SFRP2/FSTL1 (shSFRP2 and shFSTL1) were designed and constructed by Oribio (Changsha, China). OriTransPEI‐DNA (Oribio) was used to transiently transfect the plasmids into DPCs. Forty‐eight hours later, the overexpression or specific silencing effects were verified using Western blot and qRT‐PCR analyses. All sequences are listed in Table S1.

### 2.5 Enzyme‐Linked Immunosorbent Assay (ELISA) and Biochemical Assay

Using commercial kits, the expression levels of inflammatory mediators IL‐6 (CSB‐E04638h for human IL‐6, CUSABIO, Wuhan, China), IL‐1β (CSB‐E08053h for human IL‐1β, CUSABIO), TNF‐α (CSB‐E04740h for human TNF‐α, CUSABIO), and redox‐related factors MDA (BC0025, Solarbio), GSH (A006‐2‐1, Jiancheng Bioengineering Institute, Nanjing, China), and SOD (A001‐2, Jiancheng Bioengineering Institute) in human DPCs were detected. A microplate reader (5,200,110, Thermo Fisher Scientific, Waltham, USA) was employed to measure the corresponding absorbance.

### 2.6 Immunofluorescence (IF) Staining

Cells were subjected to fixation with 4% paraformaldehyde at room temperature (RT), followed by twice washing in PBS. The cell membranes were subjected to permeabilization with Triton X‐100, followed by blocking with 5% bovine serum albumin (BSA) for 1 h at RT. Subsequently, cells were incubated with primary antibodies overnight at 4°C. After washing three times with PBS, cells were incubated with fluorescent secondary antibodies for 1 h at RT in the dark, followed by PBS washing. The nuclei were stained with DAPI. A fluorescence microscope (ECLIPSE Ts2R, Nikon, Tokyo, Japan) was utilized to capture images. The following antibody information was applied: anti‐SFRP2 (1:200, 12,189‐1‐AP, Proteintech, Wuhan, China), anti‐FSTL1 (1:200, 68,528‐1‐Ig, Proteintech), Cy3 conjugated Goat Anti‐Mouse IgG (H + L) (1:200, GB21301, Servicebio, Wuhan, China), Alexa Fluor 488‐conjugated Goat Anti‐Rabbit IgG (H + L) (1:200, GB25303, Servicebio). For IF staining of skin tissues, after deparaffinizing, rehydrating and antigen retrieving, the sections were blocked with 5% BSA containing 0.3% Triton X‐100 for 1 h. Subsequently, sections were incubated with anti‐SFRP2 (1:200, 12,189‐1‐AP, Proteintech) overnight at 4°C. After washing three times with PBS, cells were incubated with Cy3 conjugated Goat Anti‐Rabbit IgG (H + L) (1:200, GB21303, Servicebio) for 1 h at RT in the dark, followed by PBS washing. The nuclei were stained with DAPI.

### 2.7 MTT Assay

Cell viability was evaluated according to the MTT kit protocol (C0009S, Beyotime, Shanghai, China). In brief, transfected DPCs were subjected to 24‐h incubation with 10 μg/mL of DHT or 0.2 mM H_2_O_2_. After treatment, 10 μL of MTT solution (5 mg/mL) was added into each well and incubated for 4 h. Then, 100 μL DMSO was added into each well to dissolve formazan crystals. The absorbance at 490 nm was measured using a microplate reader.

### 2.8 EdU Assay

The method of the kit (C0078L, Beyotime) was employed to detect cell proliferation. The cells were cultured and treated as described above in a 6‐well plate for 24 h. Then, EdU working solution (10 μM) preheated at 37°C was supplemented, followed by 2‐h incubation. Then, the culture media was discarded, cells were fixed with 4% paraformaldehyde for 15‐min at RT, rinsed in sequence, and permeabilized with 0.3% TritonX‐100. Subsequently, the Click reaction solution was supplemented, followed by 30‐min incubation at RT protected from light. DAPI was used for nucleus staining, and cells were imaged using a fluorescence microscope.

### 2.9 Wound Healing Assay

Human DPCs were cultured until the cells formed a confluent monolayer. A scratch (about 0.5–1 mm) was made on the cell monolayer perpendicular to the bottom of the culture plate. Following the scratch, cell debris was eliminated by rinsing in serum‐free media, and the cells were maintained in culture. Cell migration to the scratch area was detected using a microscope and photographed at 0 h and 48 h.

### 2.10 Transwell‐Invasion Assay

A 24‐well Transwell plate with an 8 μm pore size membrane pre‐coated with Matrigel (354,234, Corning, New York, USA) was employed to assess cell invasion. 100 μL of cell suspension (3 × 10^5^ cells) was seeded into the top chamber of the Transwell plate; after the addition of serum‐free media, 800 μL of media containing 10% FBS was supplemented into the bottom chamber. 24 h later, a cotton swab was employed to gently wipe off the non‐migrated cells within the inner layer of the chamber. Then, cells at the bottom were fixed with 4% paraformaldehyde, and then stained with 0.1% crystal violet. The number of invasive cells was counted in randomly selected microscopic fields.

### 2.11 Senescence‐Associated β‐Galactosidase (SA‐β‐Gal) Assay

Cells were subjected to 24‐h incubation with DHT within 6‐well plates. Subsequently, the media was discarded and the cells were washed in PBS. The cells were subjected to 15‐min fixation at RT with β‐galactosidase staining fixative (1 mL/well, C0602, Beyotime). The aspiration was used to discard the cell fixative, followed by washing of cells with PBS. Next, the staining working solution (1 mL/well) was supplemented, followed by an overnight incubation at 37°C. The cell staining was detected using an optical microscope and photographed.

### 2.12 Real‐Time Quantitative PCR (qRT‐PCR)

TRIzol reagent (15596018CN; Thermo Fisher Scientific) was employed to isolate total RNA from mice dorsal skin tissue or DPCs, and High‐Capacity cDNA Reverse Transcription Kit (4,368,814; Thermo Fisher Scientific) was utilized to conduct reverse transcription of RNA into cDNA. SYBR Green PCR Master Mix (4,309,155; Thermo Fisher Scientific) was applied to perform qRT‐PCR upon an ABI 7500 Real‐Time PCR system. The relative mRNA expression was assessed using the 2^−ΔΔCt^ method (sequence details provided in Table S2).

### 2.13 Flow Cytometry

The levels of mitochondrial superoxide and calcium in DPCs were detected using a Cytoflex flow cytometer (Beckman Coulter, Miami, USA) as described below. First, trypsinized DPCs were subjected to 20‐min incubation at 37°C protected from light with either 5 μM MitoSOX (M36005, Invitrogen) or 2 μM Rhod‐2 (HY‐D0989; MCE, Monmouth Junction, USA). Subsequently, after washing in PBS, cells were re‐suspended in PBS, and flow cytometry was utilized to analyze. To measure the intracellular reactive oxygen species (ROS) level, cells were rinsed with PBS, followed by 30‐min incubation at 37°C with culture media supplemented with 1 μM H2DCFDA (KGT010‐1; KeyGENE biotech, Nanjing, China). Finally, the CytoFlex (Beckman Coulter) was applied to analyze the trypsinized cells. The percentage of cells exhibiting high fluorescence intensity was determined.

### 2.14 Western Blot

Total protein samples were obtained from mouse skin tissues with hair follicles, and human DPCs using RIPA lysis buffer (P0013C; Beyotime) supplemented with protease inhibitor cocktail (P1006; Beyotime). A BCA kit (P0012; Beyotime) was employed to determine protein content, and subsequently, proteins were added to the loading buffer (P0015; Beyotime) and mixed. The mixture was subjected to 5‐min heating with a boiling water bath to denature the protein. After SDS‐PAGE electrophoresis, the separated proteins (20 μg) were electroblotted from the gel onto a PVDF membrane. The membrane was subjected to blocking with 5% skim milk within TBST, followed by an overnight incubation at 4°C with the primary antibodies: SFRP2 (1:1000, 12,189‐1‐AP; Proteintech), p21 (1:1000, ab109520; Abcam, Cambridge, UK), p16 (1:1000, bs‐23797R; Bioss, Woburn, USA), p53 (1:5000, 10,442‐1‐AP; Proteintech), PGC‐1α (1:1000, 66,369‐1‐Ig; Proteintech), FSTL1 (1:1000, 20,182‐1‐AP; Proteintech). On the following day, the membrane was rinsed with washing solution. The membrane was transferred to the secondary antibody, followed by 1‐h incubation at RT. Following the addition of the developing solution to the membrane, the chemiluminescence imaging system (Tanon‐5200; Tanon, Shanghai, China) detected the membrane.

### 2.15 Co‐Immunoprecipitation (Co‐IP)

After washing three times with ice‐cold PBS, cell lysates were harvested at 4°C using lysis buffer with protease inhibitor (Roche, Basel, Switzerland). 500 μL of lysates (200–1000 μg protein) were pre‐cleared with 5 μL of protein A/G beads for 2 h at 4°C, then incubated with 5 μg antibody (SFRP2, SFRP2‐201AP; Thermo Fisher Scientific; FSTL1, 20,182‐1‐AP, Proteintech) or 1 μg IgG overnight. The next day, samples were mixed with 5 μL beads for 3 h. The immune complex was washed 6 times and analyzed by Western blotting.

### 2.16 Differentially Expressed Genes Analysis (DEGs)

Gene expression data were obtained from GSE90594 (Michel et al. 2017), and the detection platform GPL17077 Agilent‐039494 SurePrint G3 Human GE v2 8 × 60K Microarray 039,381 (Probe Name version) was utilized to perform limma differential analysis. Gene expression data were obtained from GSE178374 (Zhang et al. 2021) and GSE212301 (Liu et al. 2022), and DESeq2 differential analysis was performed using the detection platform GPL21290 Illumina HiSeq 3000 ( *Homo sapiens* ).

### 2.17 PPI Analysis

SFRP2‐related genes were analyzed according to the online tool STRING database (https://string‐db.org/), with the construction of a PPI network.

### 2.18 Gene Ontology (GO) and Kyoto Encyclopedia of Genes and Genomes (KEGG) Enrichment Analyses

GO and KEGG enrichment analyses were conducted on the intersecting genes. The Database for Annotation, Visualization, and Integrated Discovery (DAVID, https://david.ncifcrf.gov/) and Metascape (https://metascape.org/gp/) were employed to perform these analyses. Cutoffs were set at FDR < 0.05 and *p* < 0.05. Subsequently, R version 3.4.1 was applied to visualize the results.

### 2.19 Statistical Analysis

GraphPad Prism 9.0 software (GraphPad Prism, San Diego, USA) was used to analyze all data. Before statistical testing, data distribution normality was evaluated using the Shapiro–Wilk test. For data that followed a normal distribution, comparisons among groups were assessed using one‐way analysis of variance (ANOVA) with Tukey's post hoc test, and differences between groups were assessed by Student's *t*‐test. In cases where the data did not meet the assumptions of normality, the Kruskal‐Wallis test with Dunn's post hoc test was performed. All data were presented in terms of mean ± standard deviation (SD). When the *p* value is < 0.05, the results are considered statistically significant.

## 3 Results

### 3.1 SFRP2 Is Significantly Overexpressed in DPCs of AGA Patients

**FIGURE 1:**
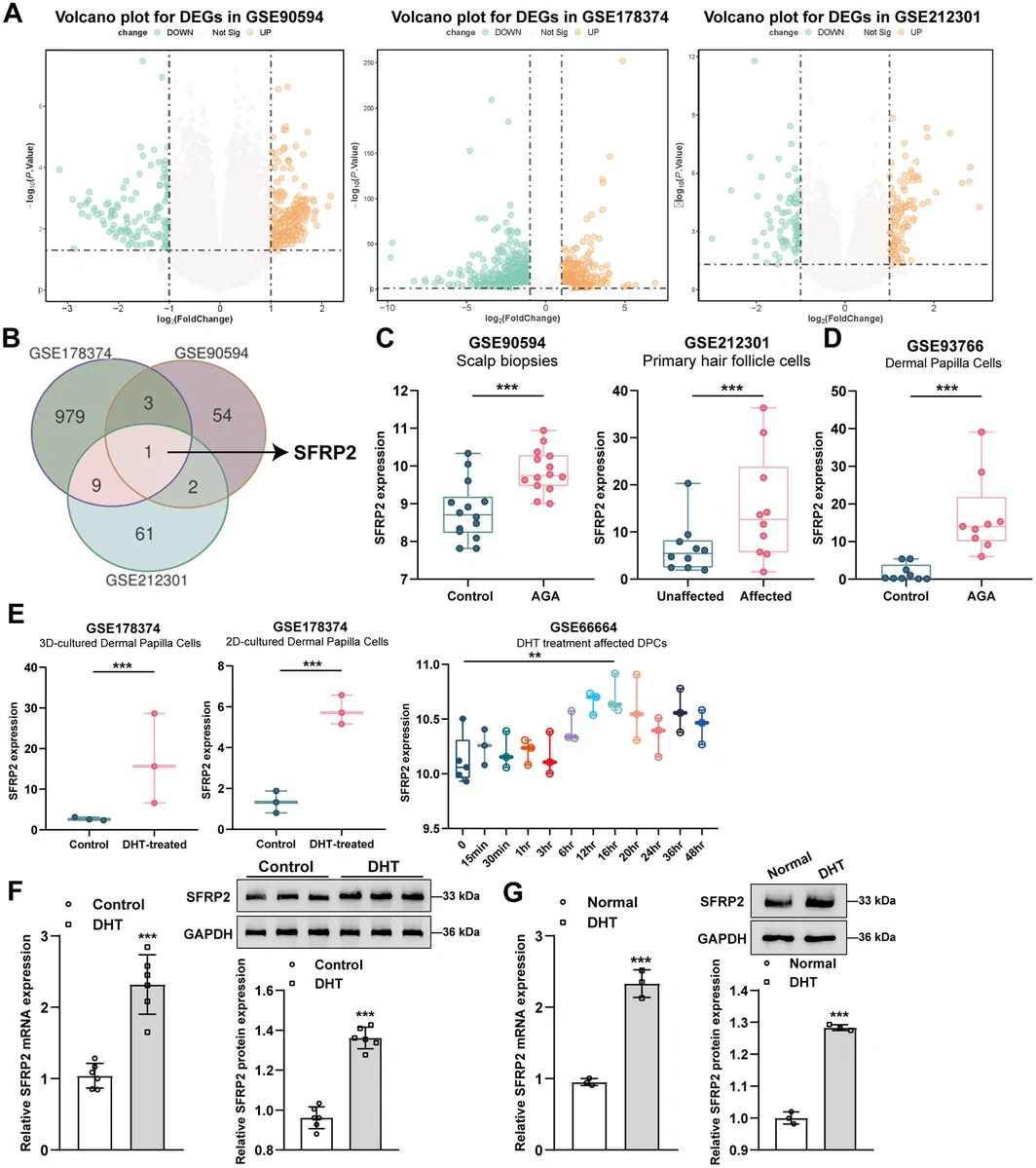
SFRP2 is significantly overexpressed in DPCs of AGA patients. (A) Based on GSE90594, GSE178374, and GSE212301 datasets, limma or DESeq2 was used to screen differentially expressed genes. (B) Venn diagram obtained by crossing three sets of data. (C) Based on chips GSE90594 and GSE212301, the expression of SFRP2 in AGA‐affected scalp, and primary hair follicle cells was analyzed. (D) Based on GSE93766, the expression of SFRP2 in human DPCs from AGA patients was analyzed. (E) Based on GSE178374 and GSE66664, the expression of SFRP2 in DHT‐treated human DPCs was analyzed. (F) Establish a DHT‐induced AGA mouse model, obtain skin tissue from the depilated area, and detect SFRP2 expression levels by qRT‐PCR and Western blot. (G) The mRNA and protein levels of SFRP2 in DHT‐treated DPCs were detected by qRT‐PCR and Western blot (F, *N* = 6; G, *N* = 3). **p < 0.01, ***p < 0.001, compared with the Control or Normal group.

To identify target genes for the treatment of AGA, we analyzed DEGs in the scalp tissue of AGA patients. By conducting differential analysis on GSE90594 (which includes scalp biopsy samples from 14 controls and 14 AGA patients), we identified 60 significantly upregulated and 246 downregulated genes in AGA samples following limma differential analysis (Figure 1A, Left). GSE178374 performed transcriptome analysis on DHT‐treated 3D‐cultured DPCs. After DESeq2 differential analysis, we found 992 significantly upregulated and 648 downregulated genes (Figure 1A, Middle). In the differential analysis by GSE212301, which compared hair follicle subsets of AGA patients (affected frontal hair follicles and unaffected occipital hair follicles), RNA‐seq data from ten paired hair follicle samples were evaluated for gene expression profiles. After DESeq2 differential analysis, 73 significantly upregulated and 116 considerably downregulated genes were identified (Figure 1A, Right). We cross‐referenced the three datasets and obtained the candidate gene SFRP2 (Figure 1B). In datasets GSE90594, GSE212301, and GSE93766, SFRP2 was significantly overexpressed in the scalp hair follicles affected by AGA (Figure 1C,D). Likewise, in dataset GSE178374, SFRP2 was highly expressed in DHT‐treated 3D‐ and 2D‐cultured DPCs (Figure 1E, Left and Middle). In dataset GSE66664 (dermal papilla cells after different time of DHT treatment), SFRP2 expression reaches a peak at 16 h (Figure 1E, Right). In response to the analysis of the dataset, this study further explored SFRP2 expression within both in vivo and in vitro models. As shown by qRT‐PCR and Western blot analyses, SFRP2 mRNA and protein expression within the DHT‐induced AGA mouse model and the DHT‐treated DPCs model were considerably higher in comparison with normal controls (Figure 1F,G). Collectively, SFRP2 is significantly overexpressed in DPCs of AGA patients.

### 3.2 SFRP2 Knockdown Improves Hair Cycle and Hair Follicle Morphology Within the DHT‐Induced AGA Model Mice

**FIGURE 2:**
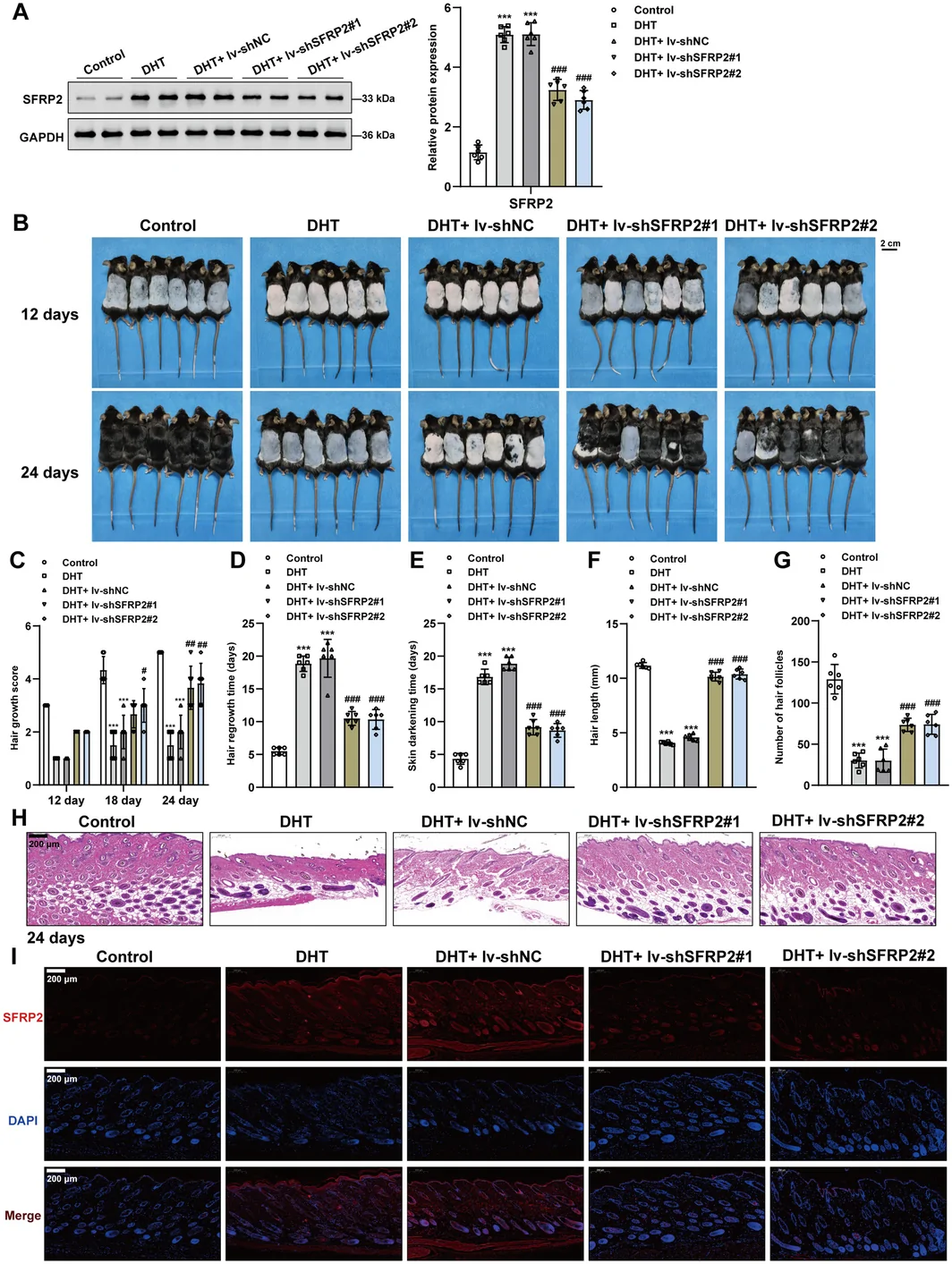
Effects of SFRP2 knockdown on hair cycle and hair follicle morphology in AGA model mice. The mice were divided into 5 groups: Control, DHT, DHT + lv‐shNC, DHT + lv‐shSFRP2#1, DHT + lv‐shSFRP2#2. (A) Western blot detection of SFRP2 protein level to verify transfection efficiency. (B) Showing photos of the back skin of mice on days 12 and 24 after hair removal. (C) Hair growth score was measured every other day. (D) Hair regrowth time. (E) Skin darkening time. (F) On the 24th day of the experiment, the average hair length of mice in each group was measured. (G) Counting the number of hair follicles based on H&E‐stained sections. (H) H&E staining to detect pathological changes of the back skin tissue, Scale bar: 200 μm. (I) Representative immunofluorescence staining of SFRP2 expression in mouse dorsal skin tissues. Scale bar = 200 μm (*N* = 6). ***p < 0.001, compared with the Control group; ^#^ *p* < 0.05, ^##^ *p* < 0.01, ^###^ *p* < 0.001, compared with the DHT + lv‐shNC group.

The effect of SFRP2 on AGA was studied using a DHT‐induced AGA model in mice, with two groups of SFRP2 shRNA lentiviral knockdown vectors (lv‐shSFRP2#1 and lv‐shSFRP2#2) being administered to the mice via the tail vein. Firstly, SFRP2 protein levels were significantly increased in the DHT group compared with the Control group, whereas lentiviral‐mediated SFRP2 knockdown markedly reduced SFRP2 protein expression, confirming efficient gene silencing at the protein level (Figure 2A). The back skins of the mice were captured on the 12th and 24th days following hair removal, indicating that mice in the Control group had fully grown hair by the 24th day. DHT‐induced mice showed slow hair growth, while the hair growth of mice treated with SFRP2 knockdown approached normal controls (Figure 2B). From the 12th day onwards, hair growth timing and skin darkening timing were recorded, and hair growth scores were assessed every 6 days. The findings demonstrated that in comparison with normal controls, the hair scores of DHT mice were lower, and both hair regeneration and skin darkening times were significantly extended. When compared with DHT + lv‐shNC mice, those treated with SFRP2 knockdown partially reversed the adverse effects of DHT (Figure 2C–E). Hair length was recorded on the 24th day, showing that hair length within DHT mice was markedly shorter in comparison with normal controls, while mice treated with SFRP2 knockdown exhibited restored hair length (Figure 2F). Next, back skin tissues were harvested from the mice for H&E staining to evaluate pathological conditions and the number of hair follicles, suggesting that the thickness of the subcutaneous tissue layer and the hair follicle number within DHT mice were remarkably reduced, while SFRP2 knockdown treatment effectively mitigated the decrease in hair follicle thickness and number caused by DHT (Figure 2G,H). Immunofluorescence staining showed that SFRP2 was mainly localized in the dermal papilla region of hair follicles. Compared with the control group, SFRP2 fluorescence intensity was markedly increased in DHT‐treated mice, whereas SFRP2 knockdown significantly reduced its expression in both lv‐shSFRP2#1 and lv‐shSFRP2#2 groups, further confirming the effective silencing of SFRP2 in vivo (Figure 2I).

### 3.3 SFRP2 Knockdown Promotes Cell Proliferation, Migration and Invasion, Anti‐Inflammation and Anti‐Senescence in DHT‐Treated DPCs In Vitro

**FIGURE 3:**
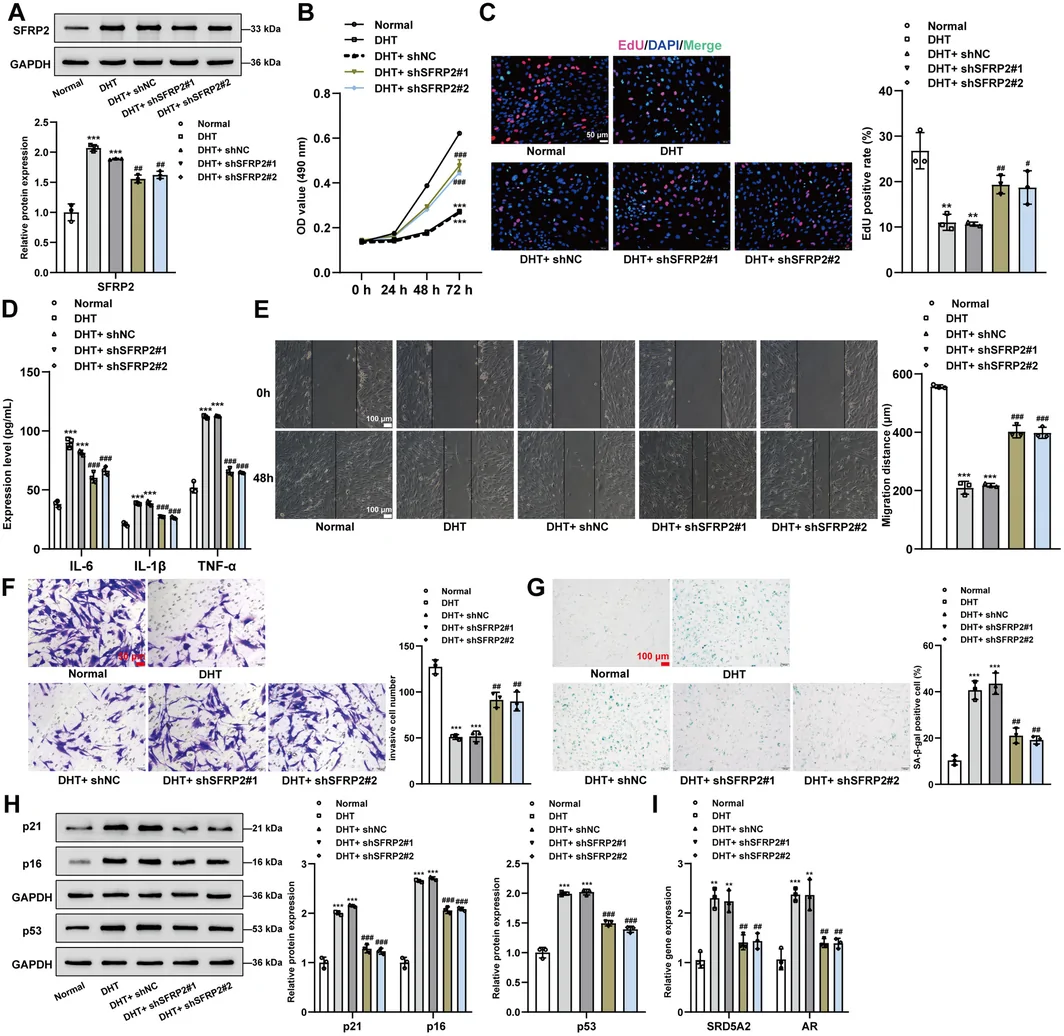
Effects of SFRP2 knockdown on proliferation, inflammation, migration and senescence of DHT‐treated human DPCs in vitro. The DPCs were divided into 5 groups: Normal, DHT, DHT + shNC, DHT + shSFRP2#1, and DHT + shSFRP2#2. (A) Western blot detection of SFRP2 protein level to verify transfection efficiency. (B) MTT assay for cell viability; (C) EdU detection of cell proliferation levels, Scale bar: 50 μm; (D) ELISA to detect the expression of inflammatory factors IL‐6, IL‐1β, TNF‐α in DPCs; (E) Wound healing assay to detect cell migration, Scale bar: 100 μm; (F) Transwell‐invasion assay to detect invasion, Scale bar: 50 μm; (G) SA‐β‐gal kit was used to detect cell senescence, Scale bar: 100 μm; (H) Western blot detection of the expression of senescence markers p21, p16 and p53; (I) QRT‐PCR detection of the expression of androgen‐related genes SRD5A2 and AR (*N* = 3). **p < 0.01, ***p < 0.001, compared with the Normal group; ^#^ *p* < 0.05, ^##^ *p* < 0.01, ^###^ *p* < 0.001, compared with the DHT + shNC group.

Next, the effect of SFRP2 upon AGA was assessed using an in vitro model in which human DPCs were treated with DHT to induce AGA‐like cellular dysfunction, where DHT is a potent androgen, leading to impaired proliferation, increased inflammatory response, mitochondrial dysfunction, and senescence, thereby mimicking the key pathological features of AGA in vitro. Similar to animal grouping, DPCs were divided into Normal, DHT, DHT + shNC, DHT + shSFRP2#1, and DHT + shSFRP2#2. SFRP2 protein levels were significantly increased in DPCs under DHT stimulation and were effectively reduced following lentiviral‐mediated SFRP2 knockdown (Figure 3A). Cell proliferation was evaluated using MTT and EdU assays, and the results showed that DHT treatment significantly reduced the proliferation of DPCs compared with the Control group, whereas SFRP2 knockdown restored DHT‐inhibited DPCs proliferation (Figure 3B,C). As revealed by ELISA, in comparison with normal controls, the release of pro‐inflammatory factors IL‐6, IL‐1β, and TNF‐α within the DHT group was increased, while SFRP2 knockdown reduced the secretion level of pro‐inflammatory factors (Figure 3D). Wound healing assay and Transwell‐invasion assay were used to assess cell migratory and invasive abilities, respectively, demonstrating that cell migratory and invasive abilities within the DHT group were reduced, and SFRP2 knockdown reversed this effect (Figure 3E,F). Next, through the SA‐β‐gal kit, it was found that the cell senescence rate within the DHT group was dramatically increased in comparison with normal controls, whereas SFRP2 knockdown partially alleviated cell senescence (Figure 3G). Western blot was conducted to detect the expression levels of cell senescence‐related proteins p21, p16 and p53. In comparison with normal controls, p21, p16 and p53 protein contents in the DHT group were upregulated, whereas SFRP2 knockdown partially reversed these changes (Figure 3H). qRT‐PCR was carried out to detect the expression levels of androgen‐associated genes SRD5A2 and AR. It was found that SRD5A2 and AR expression levels within the DHT group were increased, while SFRP2 knockdown inhibited this upregulation (Figure 3I).

### 3.4 SFRP2 Knockdown Improves Mitochondrial Dysfunction in the DHT‐Treated DPCs In Vitro

**FIGURE 4:**
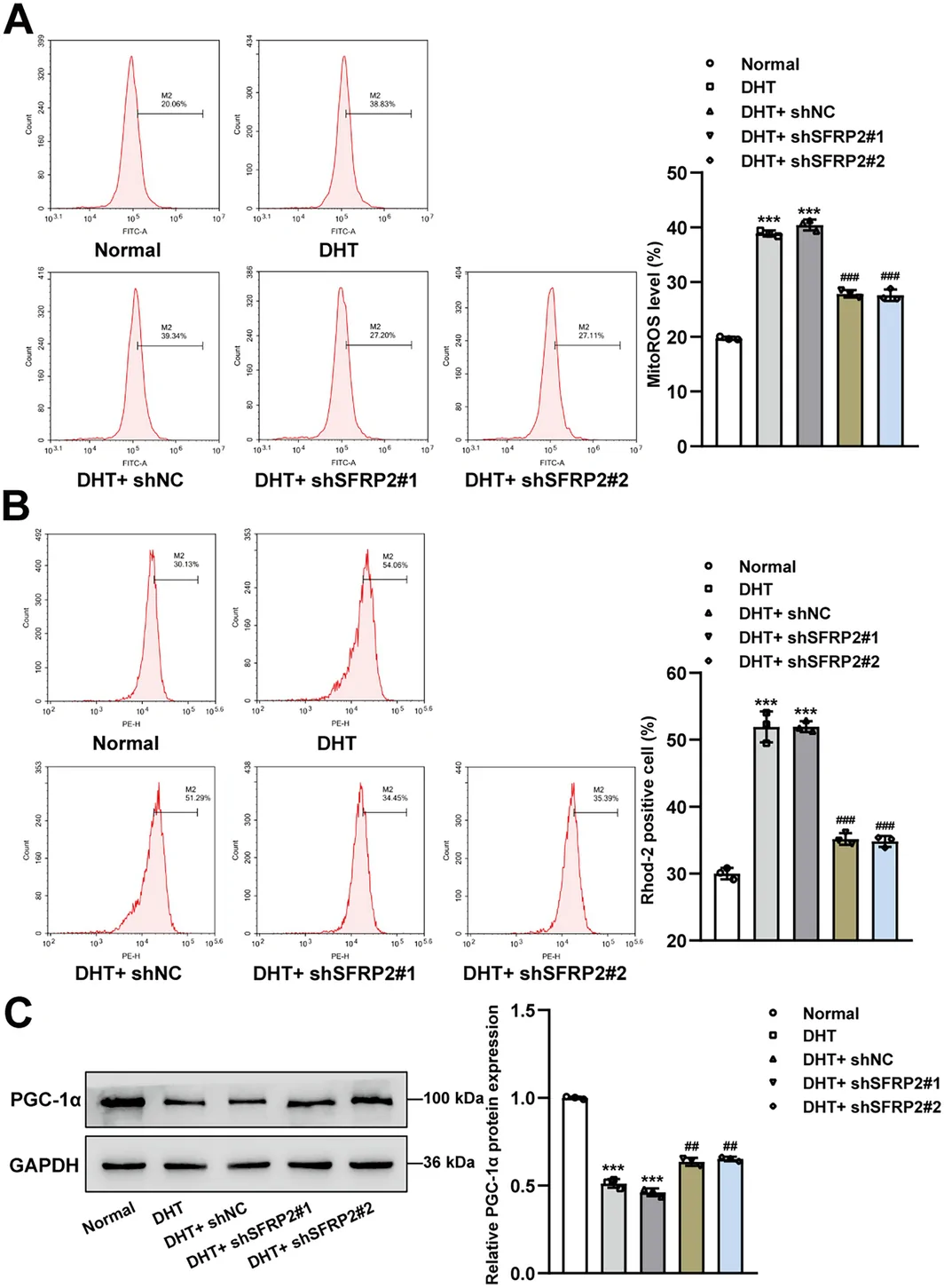
Effects of SFRP2 knockdown on mitochondrial dysfunction in DHT‐treated human DPCs in vitro. The DPCs were divided into 5 groups: Normal, DHT, DHT + shNC, DHT + shSFRP2#1, and DHT + shSFRP2#2. (A) Flow cytometry was used to detect mitochondrial reactive oxygen species (ROS) levels in DPCs after MitoSOX staining. (B) Rhod‐2 staining was used to detect mitochondrial calcium influx in DPCs. (C) Western blot detection of PGC‐1α protein expression in DPCs (*N* = 3). ***p < 0.001, compared with the Normal group; ^##^ *p* < 0.01, ^###^ *p* < 0.001, compared with the DHT + shNC group.

The effect of SFRP2 knockdown on mitochondrial dysfunction was further detected. Mitochondrial ROS levels were detected by flow cytometry after MitoSOX staining, indicating that in comparison with normal controls, the mitochondrial ROS level in the DHT‐treated DPCs was considerably elevated, and the mitochondrial ROS level in the group with SFRP2 knockdown decreased (Figure 4A). Rhod‐2 staining was used to detect mitochondrial calcium influx levels, revealing that in comparison with normal controls, the calcium influx level within the DHT group was significantly elevated, and the calcium influx level in the group with SFRP2 knockdown decreased (Figure 4B). Western blot was applied to detect the level of PGC‐1α protein, a transcriptional coenzyme activator that regulates mitochondrial antioxidants. The results showed that in comparison with normal controls, the PGC‐1α protein level within the DHT group was markedly decreased, and the PGC‐1α protein expression within the group with SFRP2 knockdown was significantly elevated (Figure 4C).

### 3.5 SFRP2 Knockdown Improves Oxidative Stress in H_2_O_2_ ‐Treated DPCs In Vitro

**FIGURE 5:**
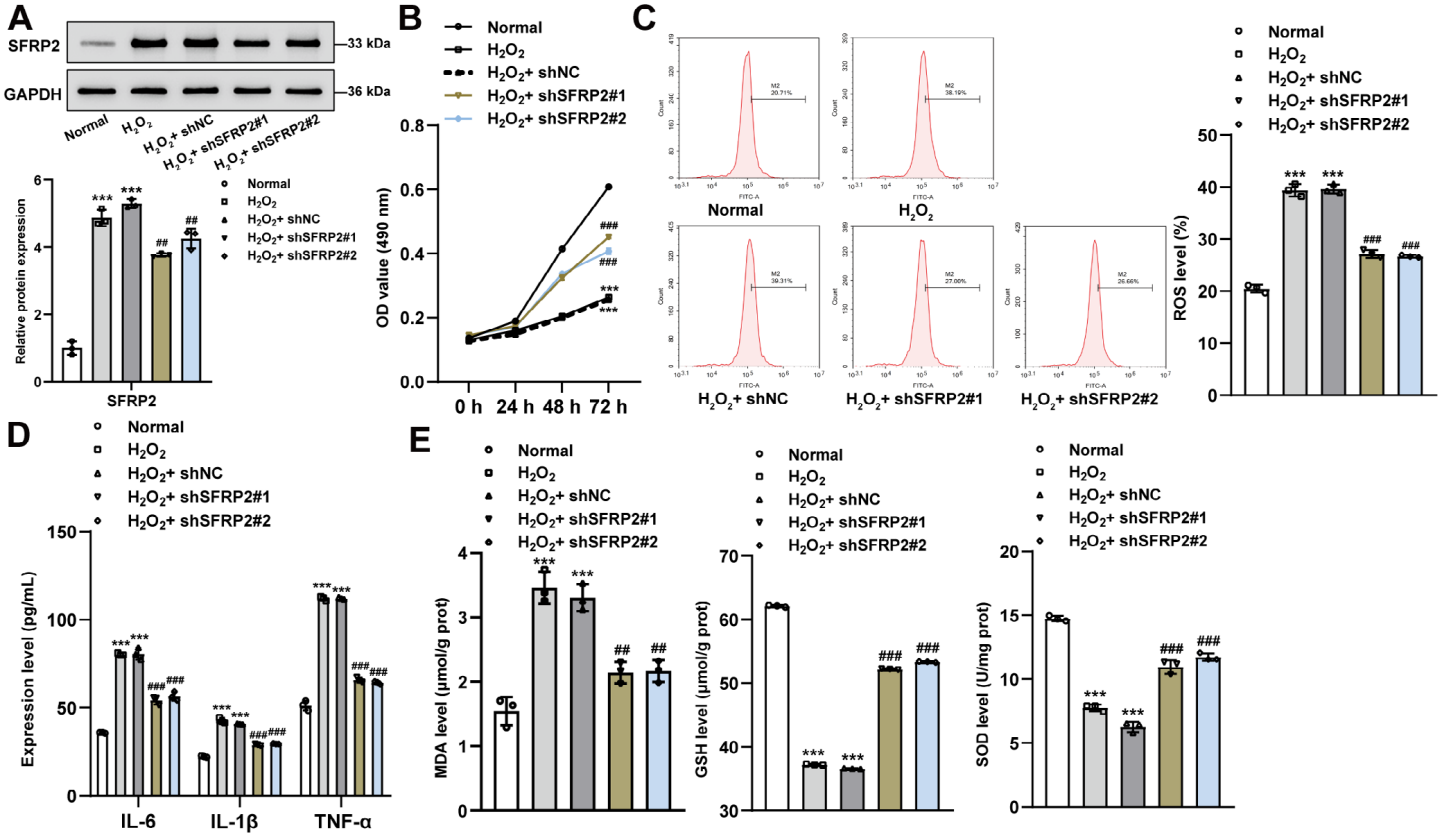
Effects of SFRP2 knockdown on oxidative stress in H_2_O_2_‐treated human DPCs in vitro. The DPCs were divided into 5 groups: Normal, H_2_O_2_, H_2_O_2_ + shNC, H_2_O_2_ + shSFRP2#1, and H_2_O_2_ + shSFRP2#2. (A) Western blot detection of SFRP2 protein level to verify transfection efficiency. (B) MTT assay for cell viability; (C) Flow cytometry was used to measure the level of intracellular ROS in DPCs; (D) ELISA to detect the expression of inflammatory factors IL‐6, IL‐1β, and TNF‐α in DPCs; (E) Biochemical kits to detect the expression of oxidative stress indicators GSH, MDA, and SOD in DPCs. (*N* = 3). ***p < 0.001, compared with the Normal group; ^##^ *p* < 0.01, ^###^ *p* < 0.001, compared with the H_2_O_2_ + shNC group.

Next, the effect of SFRP2 knockdown on oxidative stress was further detected, in which human DPCs were exposed to H_2_O_2_ to induce intracellular reactive oxygen species accumulation, thereby establishing an oxidative stress–induced cellular injury model that mimics oxidative damage and senescence observed in AGA. The DPCs were divided into 5 groups: Normal, H_2_O_2_, H_2_O_2_ + shNC, H_2_O_2_ + shSFRP2#1, and H_2_O_2_ + shSFRP2#2. SFRP2 protein expression was significantly increased in H_2_O_2_‐treated DPCs, whereas SFRP2 knockdown markedly reduced its protein levels compared with the H_2_O_2_+ shNC groups, confirming efficient silencing at the protein level (Figure 5A). MTT assay showed that cell proliferation decreased in the H_2_O_2_ group, and cell proliferation recovered after SFRP2 knockdown (Figure 5B). Flow cytometry was utilized to assess intracellular ROS levels, suggesting that intracellular ROS levels increased in the H_2_O_2_ group, and decreased after SFRP2 knockdown (Figure 5C). As shown by ELISA, the release of pro‐inflammatory mediators IL‐6, IL‐1β, TNF‐α, and lipid peroxidation product MDA increased in the H_2_O_2_ group, and the antioxidants SOD and GSH were significantly reduced. SFRP2 knockdown reversed the levels of these factors (Figure 5D,E).

### 3.6 SFRP2 Binds to Mitochondrial‐Associated Protein FSTL1

**FIGURE 6:**
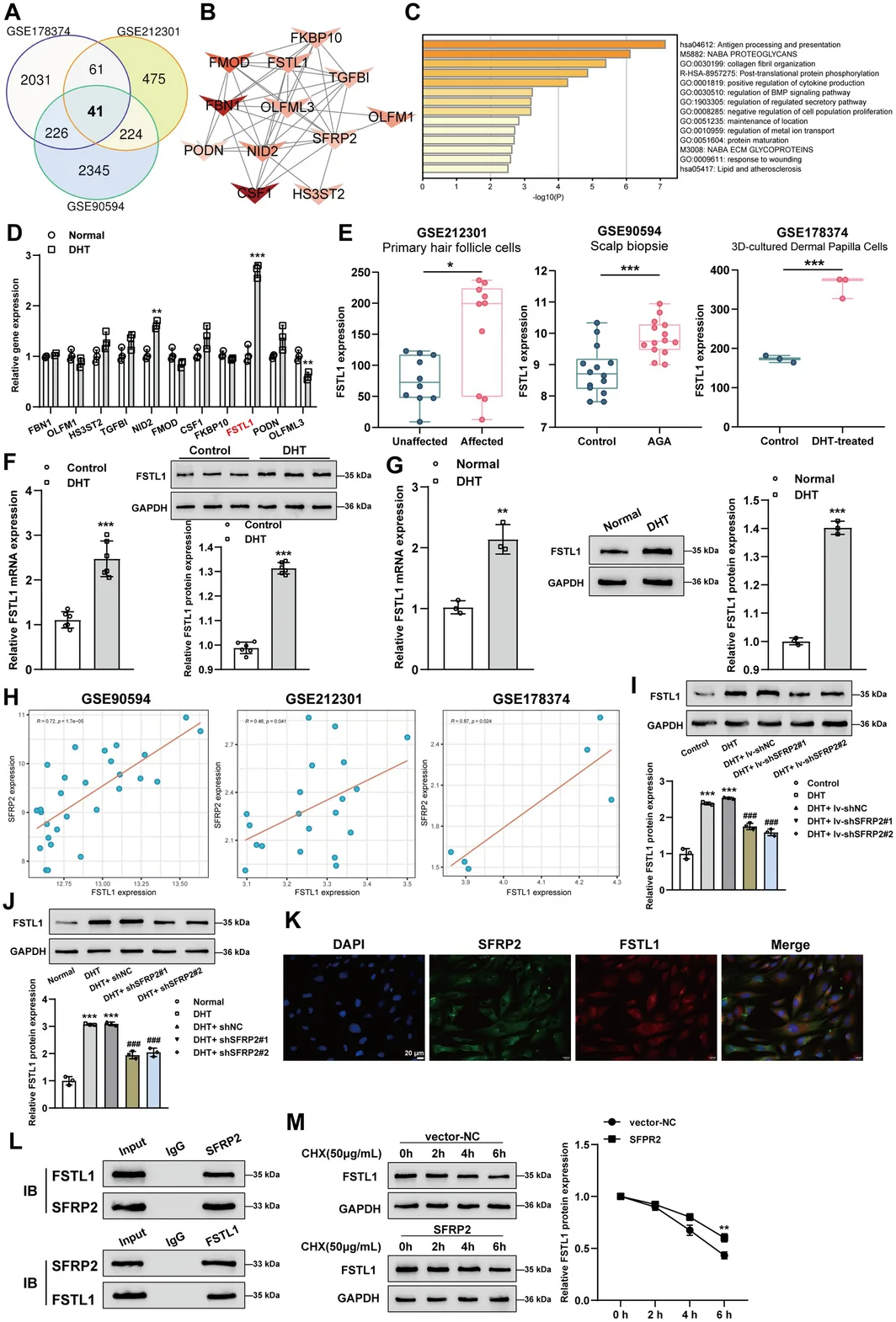
SFRP2 binds to mitochondrial‐associated protein FSTL1. (A) Screening of SFRP2‐related genes from GSE178374, GSE212301 and GSE90594; (B) The PPIs of SFRP2‐related proteins were retrieved through String (https://string‐db.org/); (C) Metascape enrichment analysis of SFRP2‐related genes; (D) QRT‐PCR detection of gene expression levels of proteins that directly interact with SFRP2 in DHT‐treated DPCs; (E) Based on GSE90594, GSE212301, and GSE178374, the expression of FSTL1 in AGA‐affected scalp, primary hair follicle cells, and 3D‐cultured DPCs was analyzed; (F) FSTL1 expression levels in the skins from control and DHT‐treated mice by qRT‐PCR and Western blot. (G) FSTL1 expression level in Normal and DHT‐treated DPCs by qRT‐PCR and Western blot. (H) Based on GSE90594, GSE212301, and GSE178374, Pearson correlation analysis was performed on the correlation between FSTL1 and SFRP2; (I) Western blot detection of the effect of knocking down SFRP2 on FSTL1 protein in the scalp tissue of AGA mouse model; (J) Western blot detection of the effect of knocking down SFRP2 on FSTL1 protein in the DHT‐treated DPCs; (K) IF detection of colocalization of SFRP2 (Green) and FSTL1 (Red) proteins, Scale bar: 20 μm; (L) Co‐immunoprecipitation (Co‐IP) assay to detect the binding of SFRP2 and FSTL1 proteins; (M) In DPCs, the protein synthesis inhibitor cycloheximide (CHX) was used to observe the effect of SFRP2 on FSTL1 protein degradation (*N* = 3). **p < 0.01, ***p < 0.001, compared with the Normal/Control/Input/vector‐NC group; *p < 0.05, ^###^ *p* < 0.001, compared with the DHT + shNC or IgG group.

SFRP2‐related genes were screened from three sets of data, GSE178374, GSE212301 and GSE90594, of which 41 genes (FSTL1, PODN, SFRP2, FXYD5, HS3ST2, PTPRJ, OLFM1, FTL, FKBP10, HSPA6, SCAMP5, SRGN, LYN, CORIN, MPP3, TNFAIP2, HLA‐B, APOL2, C3, DGKD, ABCC2, FMOD, SLC35E1, NID2, OLFML3, FXYD1, TPST1, MSC, CSF1, FLNC, DRAM1, FBN1, RILPL2, HLA‐F, DENND5A, DDIT3, IFI30, IL16, ZNFX1, BCAT1, TGFBI) exhibited a significant positive correlation with SFRP2 (Figure 6A), with *r* > 0.40, *p* < 0.05. The PPIs of these 41 proteins were further retrieved through String (https://string‐db.org/). It was found that SFRP2 could directly interact with proteins including FBN1, OLFM1, HS3ST2, TGFBI, NID2, FMOD, CSF1, FKBP10, FSTL1, PODN, and OLFML3 (Figure 6B). Metascape enrichment analysis of SFRP2‐related genes demonstrated that it was associated with processes such as Antigen processing and presentation (hsa04612), negative regulation of cell proliferation (GO:0008285), collagen fibrosis progression (GO:0030199), and wound healing (GO:0009611) (Figure 6C). qRT‐PCR detected the expression of 11 proteins that directly interacted with SFRP2 in DHT‐treated DPCs and found that FSTL1 had the highest expression level (Figure 6D). In datasets GSE212301 and GSE90594, FSTL1 was significantly overexpressed in AGA‐affected scalp hair follicles (Figure 6E, left and middle). Similarly, in dataset GSE178374, FSTL1 was highly expressed in DHT‐treated 3D cultured DPCs (Figure 6E, right). In response to the analysis of the dataset, this study further explored FSTL1 expression within both in vivo and in vitro models. As demonstrated by qRT‐PCR and Western blot, FSTL1 mRNA and protein expression within the DHT‐induced AGA mouse model and the DHT‐treated DPCs model were remarkably increased in comparison with normal controls (Figure 6F,G). Next, Pearson analysis in AGA‐related chips GSE90594 (*R* = 0.72, *p* < 0.0001), GSE212301 (*R* = 0.46, *p* = 0.041), and GSE178374 (*R* = 0.87, *p* = 0.024) revealed that SFRP2 was positively correlated with FSTL1 expression (Figure 6H). Next, the effects of SFRP2 knockdown on FSTL1 protein in vivo and in vitro models were investigated. As revealed by qRT‐PCR and Western blot analysis, FSTL1 mRNA and protein expression within the DHT‐induced AGA mouse model and the DHT‐treated DPCs model were dramatically increased in comparison with normal controls, whereas FSTL1 mRNA and protein expression were reduced after SFRP2 knockdown (Figure 6I,J). In DPCs, IF colocalization experiments found that SFRP2 and FSTL1 proteins colocalized (Figure 6K). Co‐IP experiments found that SFRP2 and FSTL1 were directly bound (Figure 6L). Finally, in DPCs, the protein synthesis inhibitor cycloheximide (CHX) was used to observe the effect of overexpression of SFRP2 upon FSTL1 protein degradation. The results showed that after 6 h of protein degradation, FSTL1 protein content within the overexpression SFRP2 group was significantly increased in comparison with the vector‐NC group, and overexpression of SFRP2 enhanced the stability of FSTL1 protein (Figure 6M).

### 3.7 SFRP2 Regulates FSTL1 to Aggravate Inflammation, Senescence and Mitochondrial Dysfunction in the DHT‐Treated DPCs In Vitro

**FIGURE 7:**
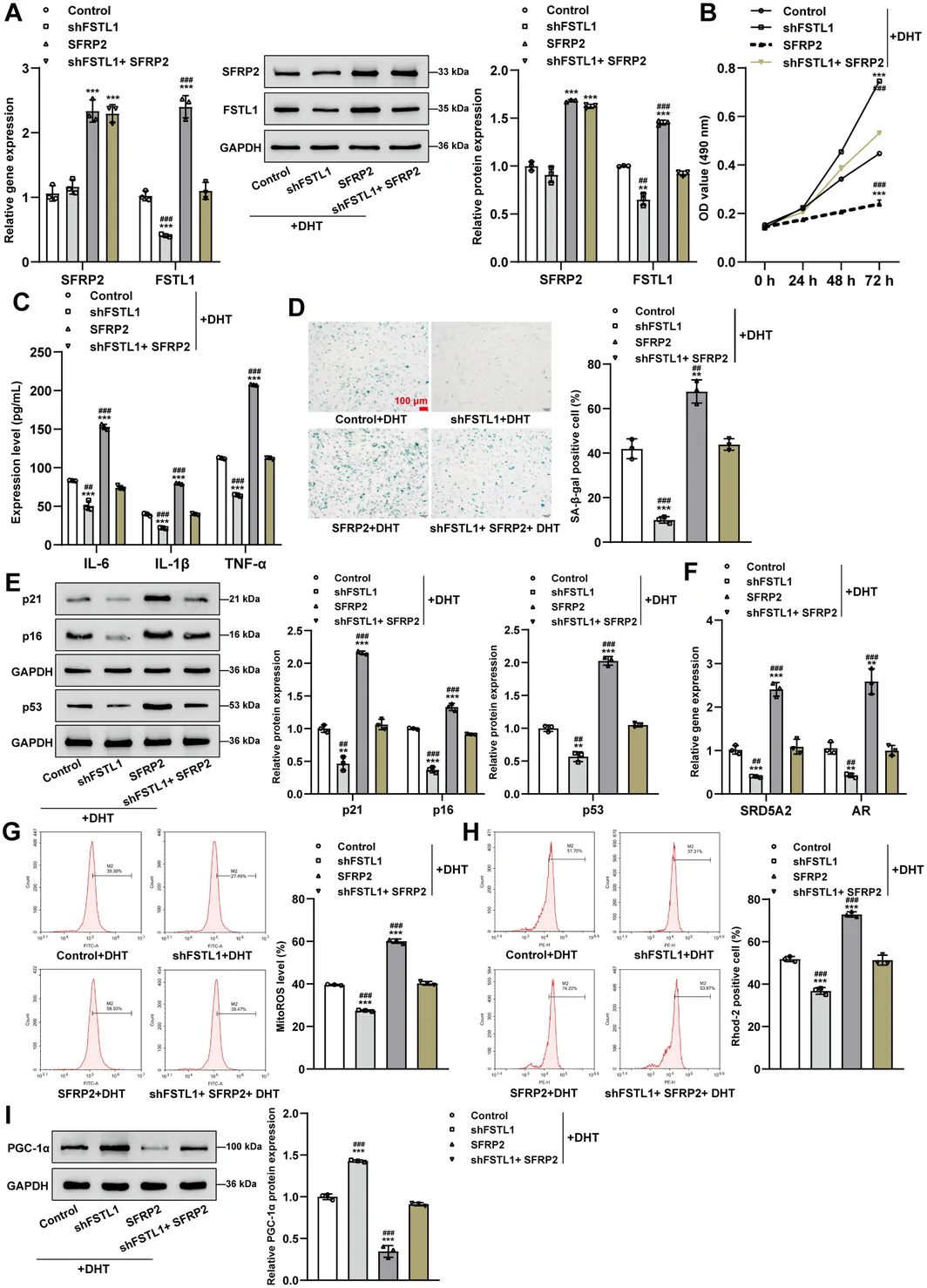
Effects of SFRP2 on FSTL1 regulation for senescence and mitochondrial dysfunction in DHT‐treated human DPCs in vitro. (A) QRT‐PCR (left) and Western blot (right) detection of transfection efficiency of SFRP2 and FSTL1. Then, DPCs were divided into 4 groups: DHT + Control (shNC+NC), DHT + shFSTL1, DHT + SFRP2, DHT + shFSTL1 + SFRP2. All groups were treated with 10 μg/mL DHT for 24 h. (B) MTT assay for cell viability; (C) ELISA to detect the expression of inflammatory factors IL‐6, IL‐1β, and TNF‐α in DPCs; (D) SA‐β‐gal kit was used to detect cell senescence, Scale bar: 100 μm; (E) Western blot detection of the expression of senescence markers p21, p16 and p53; (F) QRT‐PCR detection of the expression of androgen‐related genes SRD5A2 and AR. (G) Flow cytometry was used to detect mitochondrial ROS levels in DPCs after MitoSOX staining; (H) Rhod‐2 staining was used to detect mitochondrial calcium influx in DPCs; (I) Western blot detection of PGC‐1α protein expression in DPCs (*N* = 3). **p < 0.01, ***p < 0.001, compared with the DHT + Control/Control group; ^##^ *p* < 0.01, ^###^ *p* < 0.001, compared with the DHT + shFSTL1 + SFRP2/shFSTL1 + SFRP2 group.

The DPCs were divided into 4 groups: DHT + Control (shNC + NC), DHT + shFSTL1, DHT + SFRP2, DHT + shFSTL1 + SFRP2. All groups were subjected to 24‐h treatment with 10 μg/mL DHT. The transfection efficiency of SFRP2 and FSTL1 was detected using qRT‐PCR and Western blot, indicating that SFRP2 overexpression significantly elevated the mRNA and protein levels of SFRP2 and FSTL1, but shFSTL1 only reduced the mRNA and protein level of FSTL1 and had no effect on SFRP2 (Figure 7A). The MTT assay was used to evaluate cell proliferation, and the results showed that DHT + SFRP2 reduced cell proliferation, while knockdown of FSTL1 restored cell proliferation, and the combination of the two (DHT + shFSTL1 + SFRP2 group) offset each other (Figure 7B). The levels of pro‐inflammatory mediators were assessed using ELISA, revealing that in comparison with normal controls, the secretion of pro‐inflammatory factors IL‐6, IL‐1β and TNF‐α in the DHT + SFRP2 group increased, while knockdown of FSTL1 reduced the release level of pro‐inflammatory mediators, and the combination of the two offset each other (Figure 7C). Further anti‐aging detection was performed, and the SA‐β‐gal kit demonstrated that the cell senescence rate in the DHT + SFRP2 group increased significantly, while knockdown of FSTL1 alleviated cell senescence (Figure 7D). The expression levels of cell senescence‐associated proteins p21, p16 and p53 were evaluated using Western blot, and the results were consistent with the trend of cell senescence (Figure 7E). The expression levels of androgen‐associated genes SRD5A2 and AR were determined using qRT‐PCR, and it was found that SRD5A2 and AR expression within the DHT + SFRP2 group was increased, while FSTL1 knockdown inhibited this upregulation (Figure 7F).

Then, mitochondrial function‐related tests were conducted. The mitochondrial ROS and mitochondrial calcium influx levels were assessed using flow cytometry, suggesting that mitochondrial ROS and calcium influx levels within the DHT + SFRP2 group were significantly increased, while those in the FSTL1 knockdown group were decreased (Figure 7G,H). PGC‐1α protein contents were detected using Western blot, demonstrating that in comparison with DHT + Control group, PGC‐1α protein content within the DHT + SFRP2 group was significantly decreased, whereas that in the FSTL1 knockdown group was markedly elevated (Figure 7I). In summary, knocking down FSTL1 is beneficial for exerting anti‐inflammatory and anti‐aging effects and improving mitochondrial dysfunction, and knocking down FSTL1 can antagonize the negative effects caused by overexpression of SFRP2.

### 3.8 SFRP2 Regulates FSTL1 to Aggravate Oxidative Stress in the H_2_O_2_ ‐Treated DPCs In Vitro

**FIGURE 8:**
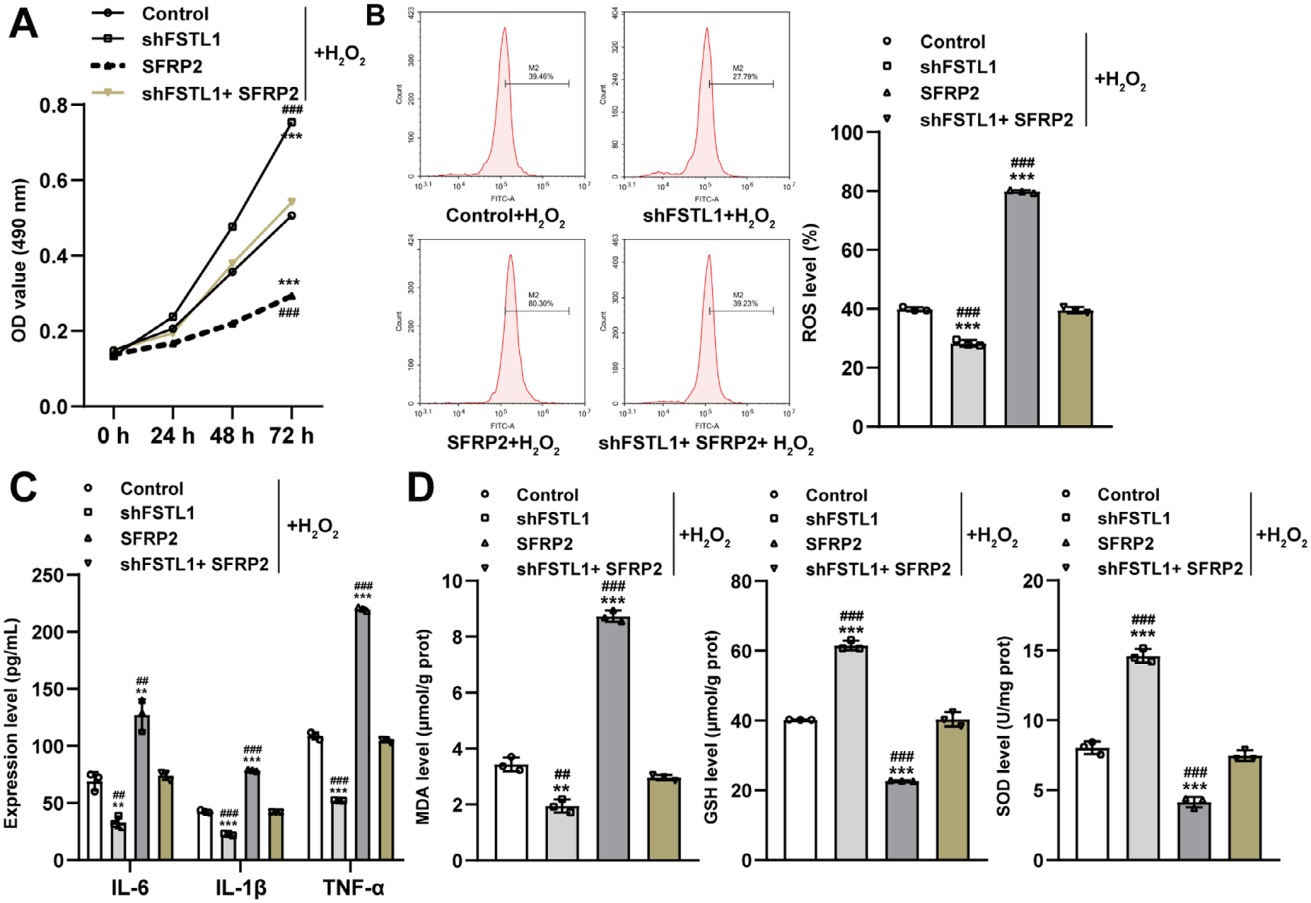
Effects of SFRP2 on FSTL1 regulation for oxidative stress in H_2_O_2_‐treated human DPCs in vitro. The DPCs were divided into 4 groups: H_2_O_2_ + Control (shNC+NC), H_2_O_2_ + shFSTL1, H_2_O_2_ + SFRP2, H_2_O_2_ + shFSTL1 + SFRP2. All groups were treated with 0.2 mM H_2_O_2_ for 24 h. (A) MTT assay for cell viability; (B) Flow cytometry was used to measure the level of intracellular ROS in DPCs; (C) ELISA to detect the expression of inflammatory factors IL‐6, IL‐1β, and TNF‐α in DPCs; (D) Biochemical kits to detect the level of oxidative stress indicators MDA, GSH, and SOD in DPCs (*N* = 3). **p < 0.01, ***p < 0.001, compared with the H_2_O_2_ + Control group; ^##^ *p* < 0.01, ^###^ *p* < 0.001, compared with the H_2_O_2_ + shFSTL1 + SFRP2 group.

The DPCs were divided into 4 groups: H_2_O_2_ + Control (shNC + NC), H_2_O_2_ + shFSTL1, H_2_O_2_ + SFRP2, H_2_O_2_ + shFSTL1 + SFRP2. All groups were subjected to 24‐h treatment with 0.2 mM H_2_O_2_. Cell proliferation was detected using the MTT assay, revealing that H_2_O_2_ + SFRP2 reduced cell proliferation, while knockdown of FSTL1 restored cell proliferation, and the combination of the two (H_2_O_2_ + shFSTL1 + SFRP2 group) offset each other (Figure 8A). Flow cytometry using H2DCFDA staining was used to detect intracellular ROS levels, indicating that the intracellular ROS level was elevated within the H_2_O_2_ + SFRP2 group, and the intracellular ROS level was reduced after FSTL1 knockdown (Figure 8B). As shown by ELISA, the release of pro‐inflammatory mediators IL‐6, IL‐1β, TNF‐α, and lipid peroxidation product MDA increased in the H_2_O_2_ + SFRP2 group, and the antioxidants SOD and GSH were significantly reduced. FSTL1 knockdown reversed the level of these factors (Figure 8C,D).

## 4 Discussion

AGA, also referred to as seborrheic alopecia, premature baldness, is a commonly seen hair disease. Several studies have shown that the onset of AGA is related to mitochondrial dynamics, oxidative stress, and cell apoptosis (Gao et al. 2023; Jung et al. 2022; Upton et al. 2015). At the same time, SFRP2 has been proven to exhibit a close association with the above biochemical processes (Ma et al. 2021). This article focuses on AGA to explore the mechanism of action of SFRP2 and FSTL1. This study has found that SFRP2 is significantly overexpressed in DPCs of AGA patients. In vivo experiments showed that knocking down SFRP2 improved the condition of hair and hair follicles in AGA mice; in vitro experiments demonstrated that knocking down SFRP2 enhanced the capacities of DPCs to proliferate, migrate, and invade, reduce inflammation, cell senescence, mitochondrial dysfunction and oxidative stress. Further studies confirmed that SFRP2 binds to FSTL1, and knocking down FSTL1 can antagonize the negative effects of SFRP2 overexpression. In summary, SFRP2 affects DPCs mitochondrial function and aging by regulating FSTL1 and is predicted to represent a new target for treating AGA.

SFRP family members are inhibitors of the Wnt signals that regulate the ability of multiple cell types to proliferate and develop (Hamida et al. 2023). SFRP2 contains a cysteine‐rich domain (CRD) similar to frizzled protein, which enables SFRP2 to specifically bind to Wnt proteins, thereby participating in the Wnt signaling regulation. This structural feature is the basis for its biological function. Through interaction with Wnt proteins, SFRP2 can affect a series of signal transduction processes in cells. Early studies have shown that SFRP2 expression increases during the degeneration of hair follicles and inhibits keratinocyte proliferation (Kim and Yoon 2014). miR‐218‐5p expression reduces SFRP2 expression, thereby activating the Wnt signaling, such as the modulation of downstream genes and the transcriptional activity of β‐catenin/T cell‐specific factors, regulating skin and hair follicle development (Zhao et al. 2019). In this study, bioinformatics screening of differentially expressed genes in AGA revealed that SFRP2 was highly expressed in the scalp or DPCs of AGA patients. DHT is an androgen transferred from testosterone by 5α‐reductase, which activates the androgen receptor in C57BL6 mice to inhibit hair regrowth, thereby simulating androgenic alopecia (Fu et al. 2021). This study further verified the high expression of SFRP2 in DHT‐induced animal and DPCs models.

FSTL1 is a secretory glycoprotein containing multiple domains (Umezu et al. 2010). Its structure is highly similar to that of follistatin, and it has characteristic cysteine‐rich domains. These structures endow FSTL1 with unique biological activities, enabling it to interact with a variety of molecules. In addition, FSTL1 participates in intercellular signaling and regulation (Horak et al. 2022; Mattiotti et al. 2018). In cervical cancer, increased FSTL1 expression significantly repressed the HeLa and C33A cells as well as their migration and invasion (Zhao et al. 2023). FSTL1 knockdown improved apoptosis, neuroinflammation, synaptic plasticity, neural oscillations and cognitive behavior within Aβ_1‐42_‐induced AD model mice (Kumari et al. 2023). FSTL1 knockdown inhibited microglial activation and alleviated depressive‐like symptoms within CUMS mice via regulating the TLR4/MyD88/NF‐κB signaling (Xiao et al. 2022). In this study, bioinformatics analysis identified FSTL1 as an SFRP2‐related gene that is significantly overexpressed in scalp hair follicles affected by AGA. This finding was further validated in DHT‐induced animal and DPCs models. The expression of SFRP2 and FSTL1 was positively correlated according to AGA‐related chips GSE90594, GSE212301, and GSE178374. In animal and cell models, knocking down SFRP2 decreased FSTL1 protein levels, and SFRP2 and FSTL1 proteins colocalized. SFRP2 can directly bind to FSTL1 and enhance its protein stability.

DHT‐induced AGA mouse presents characteristics such as hair follicle atrophy, reduced hair follicle number, and hair cycle disorder. Reportedly, Necrosulfonamide can promote the Wnt signaling activation within DPCs, promote hair growth, and improve DHT‐induced hair regrowth repression (Liu et al. 2024). Dahuang‐Gancao decoction (DGD) promotes the proliferation of hair follicle stem cells (HFSCs) by activating the Wnt/β‐catenin signaling, promoting hair follicles to enter the growth phase, thereby improving the symptoms of hair loss within AGA mice (Qian et al. 2025). The medicinal and edible plant Allium macrostemon Bunge also activates the Wnt/β‐catenin pathway to induce the release of growth factors within hair follicles (Gao et al. 2023). Since SFRP2 is an inhibitor of Wnt signaling, this study evaluated the effects of SFRP2 knockdown upon the hair cycle and hair follicle morphology within AGA model mice. After knocking down SFRP2, the thickness of hair follicles within AGA mice was restored, and the degree of atrophy was reduced. The hair follicle structure gradually became clear, effectively slowing down the reduction in the hair follicle number in AGA mice, the hair cycle gradually returned to normal, and the hair score improved. Moreover, FSTL1 has been implicated in regulating NF‐κB–associated inflammatory signaling and cellular stress responses (Chen and Liu 2019; Yan et al. 2024), which are closely linked to oxidative stress–induced senescence in DPCs (Zhang et al. 2009). NF‐κB signaling has been widely recognized as a central regulator of inflammatory and stress responses in dermal papilla cells, and its aberrant activation contributes to elevated pro‐inflammatory cytokine production (e.g., IL‐6, TNF‐α), increased oxidative stress, and premature senescence, all of which are key features observed in AGA‐associated DPCs dysfunction (Du et al. 2024). Therefore, we speculated that the SFRP2/FSTL1 axis regulates Wnt/β‐catenin and NF‐κB signaling pathways, thereby influencing dermal papilla cell proliferation, inflammatory responses, mitochondrial homeostasis, and cellular senescence, ultimately affecting hair follicle cycling and regeneration. However, the precise regulatory mechanisms by which the SFRP2/FSTL1 axis modulates Wnt/β‐catenin and NF‐κB signaling pathways require further experimental validation.

Mitochondrial dysfunction refers to a pathological state in which mitochondria are abnormal in structure or function, affecting the normal metabolism and physiological function of cells, and exerts a key effect on the initiation and development of various diseases (Li et al. 2023; Natarelli et al. 2024). In the AGA model, DHT causes excessive mitochondrial calcium accumulation and accumulation of mitochondrial ROS, leading to mitochondrial dysfunction (Jung et al. 2022). Studies have shown that the natural product Ellagic acid stimulates hair regrowth in mice and reverses DHT‐induced iron increase, lipid peroxidation and DHT‐induced mitochondrial dysfunction via the Wnt/β‐catenin signaling activation (Fu et al. 2024). Cyanidin 3‐O‐arabinoside represses DHT‐induced senescence of dermal papilla cells via regulating p38‐dependent ER‐mitochondria contacts (Jung et al. 2022). Mitochondrial dysfunction can induce DPCs aging. It has been reported that FSTL1 could accelerate the aging of nucleus pulposus cells and intervertebral disc degeneration via the TLR4/NF‐κB signaling (Yan et al. 2024). FSTL1 affects SENP1‐mediated DeSUMOylation to promote the aging of alveolar epithelial cells and worsen lung fibrogenesis (Sun et al. 2023). Herein, DHT‐induced mitochondrial dysfunction in DPCs, increased cell senescence, elevated the levels of senescence‐associated proteins p21, p16 and p53, significantly increased mitochondrial ROS levels, mitochondrial calcium influx, and significantly decreased the level of PGC‐1α protein, a transcriptional coenzyme activator regulating mitochondrial antioxidant activity. When SFRP2 or FSTL1 was knocked down, mitochondrial dysfunction was significantly improved, the cell senescence ratio decreased, p21, p16 and p53 expression was downregulated, mitochondrial ROS levels were significantly reduced, mitochondrial calcium influx was reduced, and PGC‐1α protein expression was significantly elevated. Furthermore, knocking down FSTL1 can antagonize the negative effects of SFRP2 overexpression.

Inflammation and oxidative stress cooperate through various pathways to facilitate the development of AGA. Throughout the process of AGA pathogenesis, the inflammatory response brings about changes in the microenvironment and abnormal androgen metabolism (Ketchem et al. 2023). This synergy intensifies the harm to hair follicle cells caused by oxidative stress, which in turn leads to more severe hair loss. Within the back skin of AGA mice, inflammation modifies the microenvironment of hair follicles. A large number of pro‐inflammatory factors, such as IL‐6, IL‐1β, and TNF‐α, are released, which undermine the normal physiological functions of hair follicle cells (Kong et al. 2022). In this study, both AGA model mice and DHT‐treated DPCs exhibited increased release of pro‐inflammatory factors, such as IL‐6, IL‐1β, and TNF‐α, along with elevated expression of androgen‐related genes SRD5A2 and AR. Additionally, DPCs proliferation decreased, while cell invasion and migration abilities were impaired, and oxidative stress levels increased. Knockdown of SFRP2 or FSTL1 alleviated these adverse effects, and notably, FSTL1 knockdown counteracted the detrimental effects of SFRP2 overexpression.

In this study, we systematically identified SFRP2 as a previously underexplored but functionally important regulator in AGA through integrative bioinformatics analysis and multi‐dataset validation. Unlike prior transcriptomic studies that mainly focused on inflammatory signatures or Wnt pathway components (Charoensuksira et al. 2024; Liu et al. 2022), our work is the first to demonstrate that SFRP2 is not only consistently upregulated in AGA‐related datasets but also functionally drives DPCs dysfunction under DHT stimulation. Importantly, we further reveal a novel SFRP2–FSTL1 axis, showing that SFRP2 directly interacts with and stabilizes FSTL1, thereby promoting mitochondrial dysfunction, oxidative stress, and cellular senescence. These findings extend the current understanding of SFRP2 from a Wnt pathway modulator to a key upstream regulator of androgen‐induced follicular pathology, highlighting its potential as a novel therapeutic target for AGA.

**FIGURE 9:**
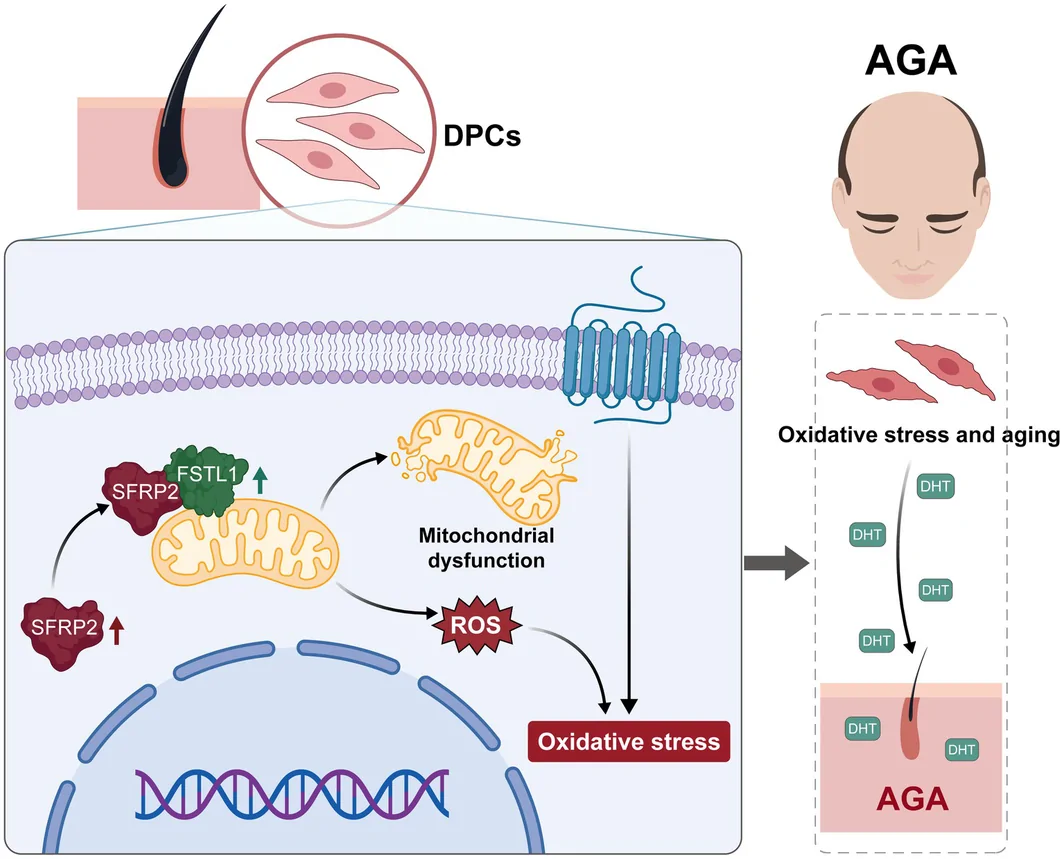
Mechanism diagram of the study showing SFRP2 regulates FSTL1 to influence DPCs mitochondrial function and cellular senescence, exerting a critical effect on AGA pathogenesis.

In conclusion, SFRP2 regulates FSTL1 to influence DPCs mitochondrial function and cellular senescence, exerting a critical effect on AGA pathogenesis (Figure 9). It holds promise as an underlying therapeutic target for AGA. Based on these findings, the present study offers a theoretical rationale and direction for the progression of effective treatment strategies.

## Author Contributions

**Youming Huang:** conceptualization, data curation, writing – original draft; **Qiong Bian:** investigation, validation, writing – original draft; **Yeyu Shen:** methodology; **Xiaoxia Ding:** resources, formal analysis; **Yan Teng:** software; **Danfeng Xu:** visualization; **Xianhong Yang:** supervision; **Yibin Fan:** funding acquisition, project administration, writing – review and editing.

## Funding

This study was supported by the Basic Research Operating Expenses of the Department of Education of Zhejiang Province (KYZD2024009), Natural Science Foundation of Zhejiang Province (LQN25H300004), Zhejiang Province Science and Technology Plan Project of Traditional Chinese Medicine (2026ZL0192), and the General Project Funds from the Health Department of Zhejiang Province (2024KY750).

## Ethics Statement

The guidelines for the care and use of animals were approved by the Medicine Animal Welfare Committee of Zhejiang Provincial People's Hospital (Approval No.: 2024‐022).

## Conflicts of Interest

The authors declare no conflicts of interest.

## Supporting information


**Table S1:** The sequences for the overexpression plasmid and shRNA plasmid construction.

**Table S2:** The primer sequences for the qRT‐PCR.

## Data Availability

The authors confirm that the data supporting the findings of this study are available within the article.

## References

[acel70666-bib-0001] Ceruti, J. M. , F. M. Oppenheimer , G. J. Leirós , and M. E. Balañá . 2021. “Androgens Downregulate BMP2 Impairing the Inductive Role of Dermal Papilla Cells on Hair Follicle Stem Cells Differentiation.” Molecular and Cellular Endocrinology 520: 111096. 10.1016/j.mce.2020.111096. 33259912

[acel70666-bib-0002] Charoensuksira, S. , S. Tantiwong , J. Pongklaokam , et al. 2024. “Disturbance of Immune Microenvironment in Androgenetic Alopecia Through Spatial Transcriptomics.” International Journal of Molecular Sciences 25, no. 16: 169031. 10.3390/ijms25169031. PMC11354591 39201715

[acel70666-bib-0003] Chen, L. , and Z. Liu . 2019. “Downregulation of FSTL‐1 Attenuates the Inflammation Injury During *Streptococcus pneumoniae* Infection by Inhibiting the NLRP3 and TLR4/NF‐κB Signaling Pathway.” Molecular Medicine Reports 20, no. 6: 5345–5352. 10.3892/mmr.2019.10752. 31638229

[acel70666-bib-0004] Chew, E. G. Y. , T. C. Lim , M. F. Leong , et al. 2022. “Observations That Suggest a Contribution of Altered Dermal Papilla Mitochondrial Function to Androgenetic Alopecia.” Experimental Dermatology 31, no. 6: 906–917. 10.1111/exd.14536. 35119146

[acel70666-bib-0005] Deng, Y. , M. Wang , Y. He , F. Liu , L. Chen , and X. Xiong . 2023. “Cellular Senescence: Ageing and Androgenetic Alopecia.” Dermatology 239, no. 4: 533–541. 10.1159/000530681. 37088073

[acel70666-bib-0006] Devjani, S. , O. Ezemma , K. J. Kelley , E. Stratton , and M. Senna . 2023. “Androgenetic Alopecia: Therapy Update.” Drugs 83, no. 8: 701–715. 10.1007/s40265-023-01880-x. 37166619 PMC10173235

[acel70666-bib-0007] Du, F. , J. Li , S. Zhang , X. Zeng , J. Nie , and Z. Li . 2024. “Oxidative Stress in Hair Follicle Development and Hair Growth: Signalling Pathways, Intervening Mechanisms and Potential of Natural Antioxidants.” Journal of Cellular and Molecular Medicine 28, no. 12: e18486. 10.1111/jcmm.18486. 38923380 PMC11196958

[acel70666-bib-0008] Ei, Z. Z. , T. Srithawirat , P. Chunhacha , et al. 2024. “Resveratrol Shows Potent Senescence Reversal in Experimental Cellular Models of Particular Matter 2.5‐Induced Cellular Senescence in Human Dermal Papilla Cells.” In Vivo 38, no. 2: 665–673. 10.21873/invivo.13487. 38418101 PMC10905444

[acel70666-bib-0009] Fu, D. , J. Huang , K. Li , et al. 2021. “Dihydrotestosterone‐Induced Hair Regrowth Inhibition by Activating Androgen Receptor in C57BL6 Mice Simulates Androgenetic Alopecia.” Biomedicine & Pharmacotherapy 137: 111247. 10.1016/j.biopha.2021.111247. 33517191

[acel70666-bib-0010] Fu, H. , W. Li , J. Liu , et al. 2024. “Ellagic Acid Inhibits Dihydrotestosterone‐Induced Ferroptosis and Promotes Hair Regeneration by Activating the Wnt/β‐Catenin Signaling Pathway.” Journal of Ethnopharmacology 330: 118227. 10.1016/j.jep.2024.118227. 38685364

[acel70666-bib-0011] Fusenig, N. E. , A. Limat , H. J. Stark , and D. Breitkreutz . 1994. “Modulation of the Differentiated Phenotype of Keratinocytes of the Hair Follicle and From Epidermis.” Journal of Dermatological Science 7, no. Suppl: S142–S151. 7999672 10.1016/0923-1811(94)90045-0

[acel70666-bib-0012] Gao, R. , Z. Yu , C. Lv , et al. 2023. “Medicinal and Edible Plant Allium Macrostemon Bunge for the Treatment of Testosterone‐Induced Androgenetic Alopecia in Mice.” Journal of Ethnopharmacology 315: 116657. 10.1016/j.jep.2023.116657. 37244409

[acel70666-bib-0013] Gupta, A. K. , M. Talukder , and G. Williams . 2022. “Comparison of Oral Minoxidil, Finasteride, and Dutasteride for Treating Androgenetic Alopecia.” Journal of Dermatological Treatment 33, no. 7: 2946–2962. 10.1080/09546634.2022.2109567. 35920739

[acel70666-bib-0014] Hamida, O. B. , M. K. Kim , and M. H. Kwack . 2023. “The Role of Dexamethasone in Mediating the Contradictory Effects of Wnt Antagonists SFRP2 and SFRP3 on Human Hair Follicle Growth.” Scientific Reports 13, no. 1: 16504. 10.1038/s41598-023-43688-5. 37783752 PMC10545675

[acel70666-bib-0015] Horak, M. , D. Fairweather , P. Kokkonen , D. Bednar , and J. Bienertova‐Vasku . 2022. “Follistatin‐Like 1 and Its Paralogs in Heart Development and Cardiovascular Disease.” Heart Failure Reviews 27, no. 6: 2251–2265. 10.1007/s10741-022-10262-6. 35867287 PMC11140762

[acel70666-bib-0016] Huang, Y. , X. Ding , Y. Shen , et al. 2026. “ *Bletilla striata* Polysaccharide (BSP) Promotes Hair Growth and Suppresses Oxidative Stress and Senescence of Dermal Papilla Cells by Inhibiting Prostaglandin‐Endoperoxide Synthase 2 (PTGS2).” International Journal of Biological Macromolecules 342, no. Pt 1: 150149. 10.1016/j.ijbiomac.2026.150149. 41519340

[acel70666-bib-0017] Jung, Y. H. , C. W. Chae , G. E. Choi , et al. 2022. “Cyanidin 3‐O‐Arabinoside Suppresses DHT‐Induced Dermal Papilla Cell Senescence by Modulating p38‐Dependent ER‐Mitochondria Contacts.” Journal of Biomedical Science 29, no. 1: 17. 10.1186/s12929-022-00800-7. 35255899 PMC8900350

[acel70666-bib-0018] Ketchem, J. M. , E. J. Bowman , and C. M. Isales . 2023. “Male Sex Hormones, Aging, and Inflammation.” Biogerontology 24, no. 1: 1–25. 10.1007/s10522-022-10002-1. 36596999 PMC9810526

[acel70666-bib-0019] Kim, B.‐K. , and S. K. Yoon . 2014. “Expression of sfrp2 Is Increased in Catagen of Hair Follicles and Inhibits Keratinocyte Proliferation.” Annals of Dermatology 26, no. 1: 79–87. 10.5021/ad.2014.26.1.79. 24648690 PMC3956799

[acel70666-bib-0020] Kong, J. , W. Qiang , J. Jiang , et al. 2022. “Safflower Oil Body Nanoparticles Deliver hFGF10 to Hair Follicles and Reduce Microinflammation to Accelerate Hair Regeneration in Androgenetic Alopecia.” International Journal of Pharmaceutics 616: 121537. 10.1016/j.ijpharm.2022.121537. 35150848

[acel70666-bib-0021] Kumari, E. , A. Xu , R. Chen , Y. Yan , Z. Yang , and T. Zhang . 2023. “FSTL1‐Knockdown Improves Neural Oscillation via Decreasing Neuronal‐Inflammation Regulating Apoptosis in Aβ1‐42 Induced AD Model Mice.” Experimental Neurology 359: 114231. 10.1016/j.expneurol.2022.114231. 36162512

[acel70666-bib-0022] Li, J. M. , X. Li , L. W. C. Chan , et al. 2023. “Lipotoxicity‐Polarised Macrophage‐Derived Exosomes Regulate Mitochondrial Fitness Through Miro1‐Mediated Mitophagy Inhibition and Contribute to Type 2 Diabetes Development in Mice.” Diabetologia 66, no. 12: 2368–2386. 10.1007/s00125-023-05992-7. 37615690

[acel70666-bib-0023] Liu, Q. , Y. Tang , Y. Huang , et al. 2022. “Insights Into Male Androgenetic Alopecia Using Comparative Transcriptome Profiling: Hypoxia‐Inducible Factor‐1 and Wnt/β‐Catenin Signalling Pathways.” British Journal of Dermatology 187, no. 6: 936–947. 10.1111/bjd.21783. 35862273 PMC10087000

[acel70666-bib-0024] Liu, Y. , S. Yang , L. Tan , et al. 2024. “Necrosulfonamide Promotes Hair Growth and Ameliorates DHT‐Induced Hair Growth Inhibition.” Journal of Dermatological Science 115, no. 2: 64–74. 10.1016/j.jdermsci.2024.04.004. 39043505

[acel70666-bib-0025] Ly, N. Y. , S. Fruechte , M. K. Hordinsky , N. Sadick , S. Arruda , and R. S. Farah . 2023. “Medical and Procedural Treatment of Androgenetic Alopecia ‐ Where Are We?” Journal of the American Academy of Dermatology 89, no. 2S: S36–S39. 10.1016/j.jaad.2023.05.004. 37591565

[acel70666-bib-0026] Ma, L. , H. Shen , C. Fang , T. Chen , and J. Wang . 2022. “Camellia Seed Cake Extract Supports Hair Growth by Abrogating the Effect of Dihydrotestosterone in Cultured Human Dermal Papilla Cells.” Molecules 27, no. 19: 6443. 10.3390/molecules27196443. 36234980 PMC9572183

[acel70666-bib-0027] Ma, T. , X. Huang , H. Zheng , et al. 2021. “SFRP2 Improves Mitochondrial Dynamics and Mitochondrial Biogenesis, Oxidative Stress, and Apoptosis in Diabetic Cardiomyopathy.” Oxidative Medicine and Cellular Longevity 2021: 9265016. 10.1155/2021/9265016. 34790288 PMC8592716

[acel70666-bib-0028] Mattiotti, A. , S. Prakash , P. Barnett , and M. J. B. van den Hoff . 2018. “Follistatin‐Like 1 in Development and Human Diseases.” Cellular and Molecular Life Sciences: CMLS 75, no. 13: 2339–2354. 10.1007/s00018-018-2805-0. 29594389 PMC5986856

[acel70666-bib-0029] Michel, L. , P. Reygagne , P. Benech , et al. 2017. “Study of Gene Expression Alteration in Male Androgenetic Alopecia: Evidence of Predominant Molecular Signalling Pathways.” British Journal of Dermatology 177, no. 5: 1322–1336. 10.1111/bjd.15577. 28403520

[acel70666-bib-0030] Natarelli, N. , N. Gahoonia , S. Aflatooni , S. Bhatia , and R. K. Sivamani . 2024. “Dermatologic Manifestations of Mitochondrial Dysfunction: A Review of the Literature.” International Journal of Molecular Sciences 25, no. 6: 3303. 10.3390/ijms25063303. 38542277 PMC10970650

[acel70666-bib-0031] Nestor, M. S. , G. Ablon , A. Gade , H. Han , and D. L. Fischer . 2021. “Treatment Options for Androgenetic Alopecia: Efficacy, Side Effects, Compliance, Financial Considerations, and Ethics.” Journal of Cosmetic Dermatology 20, no. 12: 3759–3781. 10.1111/jocd.14537. 34741573 PMC9298335

[acel70666-bib-0032] Philpott, M. 2022. “Transcriptomic Analysis Identifies Regulators of the Wnt Signalling and Hypoxia‐Inducible Factor Pathways as Possible Mediators of Androgenetic Alopecia.” British Journal of Dermatology 187, no. 6: 845. 10.1111/bjd.21881. 36199226 PMC10092041

[acel70666-bib-0033] Qian, H. , Z. Ye , Y. Hu , et al. 2025. “Dahuang‐Gancao Decoction Ameliorates Testosterone‐Induced Androgenetic Alopecia in Mice.” Journal of Ethnopharmacology 341: 119347. 10.1016/j.jep.2025.119347. 39800247

[acel70666-bib-0034] Shin, J. Y. , J. Kim , Y.‐H. Choi , N.‐G. Kang , and S. Lee . 2021. “Dexpanthenol Promotes Cell Growth by Preventing Cell Senescence and Apoptosis in Cultured Human Hair Follicle Cells.” Current Issues in Molecular Biology 43, no. 3: 1361–1373. 10.3390/cimb43030097. 34698060 PMC8929036

[acel70666-bib-0035] Sun, W. , X. Yang , L. Chen , et al. 2023. “FSTL1 Promotes Alveolar Epithelial Cell Aging and Worsens Pulmonary Fibrosis by Affecting SENP1‐Mediated DeSUMOylation.” Cell Biology International 47, no. 10: 1716–1727. 10.1002/cbin.12062. 37369969

[acel70666-bib-0036] Taghiabadi, E. , M. A. Nilforoushzadeh , and N. Aghdami . 2020. “Maintaining Hair Inductivity in Human Dermal Papilla Cells: A Review of Effective Methods.” Skin Pharmacology and Physiology 33, no. 5: 280–292. 10.1159/000510152. 33053562

[acel70666-bib-0037] Trüeb, R. M. 2021. “Oxidative Stress and Its Impact on Skin, Scalp and Hair.” International Journal of Cosmetic Science 43, no. Suppl 1: 12736. 10.1111/ics.12736. 34424547

[acel70666-bib-0038] Umezu, T. , H. Yamanouchi , Y. Iida , M. Miura , and Y. Tomooka . 2010. “Follistatin‐Like‐1, a Diffusible Mesenchymal Factor Determines the Fate of Epithelium.” Proceedings of the National Academy of Sciences of the United States of America 107, no. 10: 4601–4606. 10.1073/pnas.0909501107. 20176958 PMC2842048

[acel70666-bib-0039] Upton, J. H. , R. F. Hannen , A. W. Bahta , N. Farjo , B. Farjo , and M. P. Philpott . 2015. “Oxidative Stress‐Associated Senescence in Dermal Papilla Cells of Men With Androgenetic Alopecia.” Journal of Investigative Dermatology 135, no. 5: 1244–1252. 10.1038/jid.2015.28. 25647436

[acel70666-bib-0040] van Loon, K. , E. J. M. Huijbers , and A. W. Griffioen . 2021. “Secreted Frizzled‐Related Protein 2: A Key Player in Noncanonical Wnt Signaling and Tumor Angiogenesis.” Cancer Metastasis Reviews 40, no. 1: 191–203. 10.1007/s10555-020-09941-3. 33140138 PMC7897195

[acel70666-bib-0041] Xiao, X. , H. Zhang , W. Ning , Z. Yang , Y. Wang , and T. Zhang . 2022. “Knockdown of FSTL1 Inhibits Microglia Activation and Alleviates Depressive‐Like Symptoms Through Modulating TLR4/MyD88/NF‐κB Pathway in CUMS Mice.” Experimental Neurology 353: 114060. 10.1016/j.expneurol.2022.114060. 35367454

[acel70666-bib-0042] Yan, X. , J. Y. Ding , R. J. Zhang , et al. 2024. “FSTL1 Accelerates Nucleus Pulposus Cell Senescence and Intervertebral Disc Degeneration Through TLR4/NF‐κB Pathway.” Inflammation 47, no. 4: 1229–1247. 10.1007/s10753-024-01972-0. 38316670

[acel70666-bib-0043] Zhang, H.‐L. , X.‐X. Qiu , and X.‐H. Liao . 2024. “Dermal Papilla Cells: From Basic Research to Translational Applications.” Biology 13, no. 10: 842. 10.3390/biology13100842. 39452150 PMC11504027

[acel70666-bib-0044] Zhang, Y. , J. Huang , D. Fu , et al. 2021. “Transcriptome Analysis Reveals an Inhibitory Effect of Dihydrotestosterone‐Treated 2D‐ and 3D‐Cultured Dermal Papilla Cells on Hair Follicle Growth.” Frontiers in Cell and Developmental Biology 9: 724310. 10.3389/fcell.2021.724310. 34604224 PMC8484716

[acel70666-bib-0045] Zhang, Y. , P. Tomann , T. Andl , et al. 2009. “Reciprocal Requirements for EDA/EDAR/NF‐kappaB and Wnt/Beta‐Catenin Signaling Pathways in Hair Follicle Induction.” Developmental Cell 17, no. 1: 49–61. 10.1016/j.devcel.2009.05.011. 19619491 PMC2859042

[acel70666-bib-0046] Zhao, B. , Y. Chen , N. Yang , et al. 2019. “miR‐218‐5p Regulates Skin and Hair Follicle Development Through Wnt/β‐Catenin Signaling Pathway by Targeting SFRP2.” Journal of Cellular Physiology 234, no. 11: 20329–20341. 10.1002/jcp.28633. 30953362

[acel70666-bib-0047] Zhao, C. , Z. Chen , L. Zhu , et al. 2023. “The BMP Inhibitor Follistatin‐Like 1 (FSTL1) Suppresses Cervical Carcinogenesis.” Frontiers in Oncology 13: 1100045. 10.3389/fonc.2023.1100045. 36756161 PMC9901576

[acel70666-bib-0048] Zheng, W. , and C.‐H. Xu . 2023. “Innovative Approaches and Advances for Hair Follicle Regeneration.” ACS Biomaterials Science & Engineering 9, no. 5: 2251–2276. 10.1021/acsbiomaterials.3c00028. 37036820

