# Supplementary material for: Secreted Frizzled‐Related Protein 2 (SFRP2) Induces Follistatin‐Like 1 (FSTL1) to Regulate Dihydrotestosterone (DHT)‐Induced Dermal Papilla Cell Mitochondrial Dysfunction and Senescence
Source: Aging Cell. 2026 Sep 4;25(9):e70666. doi: 10.1111/acel.70666 (PMC13545508; doi:10.1111/acel.70666)
Supplement: Supplementary file 1 [file ACEL-25-e70666-s001.docx]

**Table S1. The sequences for the overexpression plasmid and shRNA plasmid construction**

| **Gene** | **Primer** | **Sequence** **(5'-3')** |
| --- | --- | --- |
| ShNC (hsa) | Forward | GATCCGCAGATGAAGGCACGGTCACGCTCGAGCGTGACCGTGCCTTCATCTGCTTTTTT |
|  | Reverse | CTAGAAAAAAGCAGATGAAGGCACGGTCACGCTCGAGCGTGACCGTGCCTTCATCTGCG |
| shSFRP2#1 (hsa) | Forward | GATCCCCGAAAGGGACCTGAAGAAATCTCGAGATTTCTTCAGGTCCCTTTCGGTTTTTT |
|  | Reverse | CTAGAAAAAACCGAAAGGGACCTGAAGAAATCTCGAGATTTCTTCAGGTCCCTTTCGGG |
| shSFRP2#2 (hsa) | Forward | GATCCCAAAGGTATGTGAAGCCTGCACTCGAGTGCAGGCTTCACATACCTTTGTTTTTT |
|  | Reverse | CTAGAAAAAACAAAGGTATGTGAAGCCTGCACTCGAGTGCAGGCTTCACATACCTTTGG |
| Vector-NC (hsa) | Forward | / |
|  | Reverse | / |
| SFRP2 (hsa) | Forward | GGACTCAGATCTCGAGGCCACCATGCTGCAGGGCCCTGGC |
|  | Reverse | TAGAGTCGCGGGATCCCTAGCACTGCAGCTTGCGG |
| shFSTL1 (hsa) | Forward | GATCCGATTGGAAACTCAGCTTCCAACTCGAGTTGGAAGCTGAGTTTCCAATCTTTTTT |
|  | Reverse | CTAGAAAAAAGATTGGAAACTCAGCTTCCAACTCGAGTTGGAAGCTGAGTTTCCAATCG |
| shFSTL1 (hsa) | Forward | GATCCGCTAAGGAGCAAATCCAAGATCTCGAGATCTTGGATTTGCTCCTTAGCTTTTTT |
|  | Reverse | CTAGAAAAAAGCTAAGGAGCAAATCCAAGATCTCGAGATCTTGGATTTGCTCCTTAGCG |
| lv-shNC  (mmu) | Forward | GATCCGCAGATGAAGGCACGGTCACGCTCGAGCGTGACCGTGCCTTCATCTGCTTTTTT |
|  | Reverse | CTAGAAAAAAGCAGATGAAGGCACGGTCACGCTCGAGCGTGACCGTGCCTTCATCTGCG |
| lv-shSFRP2#1  (mmu) | Forward | AATTCCGGCATCGAGTACCAGAACATCTCGAGATGTTCTGGTACTCGATGCCGTTTTTA |
|  | Reverse | CGCGTAAAAACGGCATCGAGTACCAGAACATCTCGAGATGTTCTGGTACTCGATGCCGG |
| lv-shSFRP2#2(mmu) | Forward | AATTCCCAGAGAGAGTTCAAGCGCATCTCGAGATGCGCTTGAACTCTCTCTGGTTTTTA |
|  | Reverse | CGCGTAAAAACCAGAGAGAGTTCAAGCGCATCTCGAGATGCGCTTGAACTCTCTCTGGG |
