# Supplementary material for: Secreted Frizzled‐Related Protein 2 (SFRP2) Induces Follistatin‐Like 1 (FSTL1) to Regulate Dihydrotestosterone (DHT)‐Induced Dermal Papilla Cell Mitochondrial Dysfunction and Senescence
Source: Aging Cell. 2026 Sep 4;25(9):e70666. doi: 10.1111/acel.70666 (PMC13545508; doi:10.1111/acel.70666)
Supplement: Supplementary file 2 [file ACEL-25-e70666-s002.docx]

**Table S2. The primer sequences for the qRT-PCR**

| **Gene** | **Primer** | **Sequence** **(5'-3')** |
| --- | --- | --- |
| Sfrp2 (mmu) | Forward | CCCCTGTCTGTCTCGACGA |
|  | Reverse | GTCGCACTCCAGCATGTCT |
| Fstl1 (mmu) | Forward | TCGTGGAGCAGAATGAAACAGC |
|  | Reverse | TCTCAGGAGGGTTGAAGGATGG |
| SFRP2 (hsa) | Forward | AACCTACATCAACCGAGATACCA |
|  | Reverse | CTTCAGGTCCCTTTCGGACAC |
| AR (hsa) | Forward | CCAGGGACCATGTTTTGCC |
|  | Reverse | CGAAGACGACAAGATGGACAA |
| SRD5A2 (hsa) | Forward | CGGTTTAGCTTGGGTGTCTTC |
|  | Reverse | CCGAGGAAATTGGCTCCAGAA |
| NID2 (hsa) | Forward | GAAACGCAGTATGTGGACTATGA |
|  | Reverse | GGAGGTGTCCTCTCGGTACAG |
| OLFM1 (hsa) | Forward | AGGGCAGGTGTATCTGCAC |
|  | Reverse | CCGTGAACACATGGTCTGCT |
| HS3ST2 (hsa) | Forward | CCTCTCCGGTTCCAACCAC |
|  | Reverse | GTACTCGGATAAACTCCAGCAC |
| FMOD (hsa) | Forward | GTTCCCTCCCGCATGAAGTAT |
|  | Reverse | TGGCATTGTCAAAGACGCCT |
| TGFBI (hsa) | Forward | ATGACCCTCACCTCTATGTACC |
|  | Reverse | CACAGTTCACAGTTACAATCCCA |
| CSF1 (hsa) | Forward | AGACCTCGTGCCAAATTACATT |
|  | Reverse | AGGTGTCTCATAGAAAGTTCGGA |
| FKBP10 (hsa) | Forward | AAGGACGCTGTCCAGCTAGA |
|  | Reverse | GTAGTGGTAGCGCATGAAGTC |
| PODN (hsa) | Forward | GGCTGCCTCGAAATGTCCAT |
|  | Reverse | GGTACAGGTACTCAAGTGACTCG |
| OLFML3 (hsa) | Forward | GCGGGAGGTAGACTATCTGGA |
|  | Reverse | CTTGAGAGATTGTGTAGCCACAG |
| FBN1 (hsa) | Forward | TTTAGCGTCCTACACGAGCC |
|  | Reverse | CCATCCAGGGCAACAGTAAGC |
| FSTL1 (hsa) | Forward | CTCTAAAGGCAGCAACTACAGTG |
|  | Reverse | CCCCTAAGCAACTTGTTGTTCTC |
